# Reduced expression of mitochondrial complex I subunit *Ndufs2* does not impact healthspan in mice

**DOI:** 10.1038/s41598-022-09074-3

**Published:** 2022-03-25

**Authors:** Gregory S. McElroy, Ram P. Chakrabarty, Karis B. D’Alessandro, Yuan-Shih Hu, Karthik Vasan, Jerica Tan, Joshua S. Stoolman, Samuel E. Weinberg, Elizabeth M. Steinert, Paul A. Reyfman, Benjamin D. Singer, Warren C. Ladiges, Lin Gao, José Lopéz-Barneo, Karen Ridge, G. R. Scott Budinger, Navdeep S. Chandel

**Affiliations:** 1 Department of Medicine Division of Pulmonary and Critical Care Medicine, Northwestern University Feinberg School of Medicine, Chicago, IL USA; 2 Department of Pathology, Northwestern University Feinberg School of Medicine, Chicago, IL USA; 3 Department of Comparative Medicine, University of Washington School of Medicine, Seattle, WA USA; 4 Instituto de Biomedicina de Sevilla (IBiS), Hospital Universitario Virgen del Rocío, CSIC, Universidad de Sevilla, Seville, Spain; 5 Departamento de Fisiología Médica y Biofísica, Facultad de Medicina, Universidad de Sevilla, Seville, Spain; 6 Centro de Investigación Biomédica en Red Sobre Enfermedades Neurodegenerativas (CIBERNED), Madrid, Spain; 7 Department of Biochemistry and Molecular Genetics, Northwestern University Feinberg School of Medicine, Chicago, IL 60611 USA

**Keywords:** Ageing, Mouse

## Abstract

Aging in mammals leads to reduction in genes encoding the 45-subunit mitochondrial electron transport chain complex I. It has been hypothesized that normal aging and age-related diseases such as Parkinson’s disease are in part due to modest decrease in expression of mitochondrial complex I subunits. By contrast, diminishing expression of mitochondrial complex I genes in lower organisms increases lifespan. Furthermore, metformin, a putative complex I inhibitor, increases healthspan in mice and humans. In the present study, we investigated whether loss of one allele of *Ndufs2*, the catalytic subunit of mitochondrial complex I, impacts healthspan and lifespan in mice. Our results indicate that *Ndufs2* hemizygous mice (*Ndufs2*^+/−^) show no overt impairment in aging-related motor function, learning, tissue histology, organismal metabolism, or sensitivity to metformin in a C57BL6/J background. Despite a significant reduction of *Ndufs2* mRNA, the mice do not demonstrate a significant decrease in complex I function. However, there are detectable transcriptomic changes in individual cell types and tissues due to loss of one allele of *Ndufs2*. Our data indicate that a 50% decline in mRNA of the core mitochondrial complex I subunit *Ndufs2* is neither beneficial nor detrimental to healthspan.

## Introduction

Significant progress has been made in characterizing the molecular events that are common in aging. Some of these pathways have been targeted genetically or pharmacologically in mice to determine causality^1–5^. One of the most consistent observations in aging animals is a decline in mitochondrial gene expression^6,7^. In addition to the observed expression changes, mild mitochondrial functional impairment is observed in aging tissues and is associated with numerous aging-associated diseases. Examples include neurodegenerative diseases like Parkinson’s disease, sarcopenia, and pulmonary diseases^8–13^. Mitochondrial metabolism sits in the center of a nexus of cellular processes that have been associated with aging^14–19^. Furthermore, a recent parabiosis study in mice concluded that young blood invokes rescue of the decline in age associated genes encoding electron transport chain subunits suggesting a key role of mitochondrial function in parabiosis-mediated rejuvenation^20^. This has led to the inclusion of mitochondrial dysfunction as one of the cellular hallmarks of aging^21^.

Mutations in genes encoding mitochondrial proteins that lead to severe decreases in mitochondrial function cause dramatic multi-system pathology and a shortened lifespan in model organisms and in human patients with mitochondrial diseases^22^. For example RNAi knockdown of ETC complex I subunit ND-49 in *D. melanogaster* leads to TOR pathway activation and reduced survival^23^. Paradoxically, however, in a landmark study in *C. elegans,* knockdown of the ETC and ATP synthase with RNAi during development resulted in an increase in lifespan^24^. Numerous additional studies have indicated that knockdown or mutation of components of the ETC in *C. elegans* and *D. melanogaster* can have a paradoxical significant life extending effect^25–34^. Contrasting these paradoxical life extending results, vertebrate models with genetically altered expression of ETC components have generally demonstrated detrimental effects on lifespan and healthspan with few exceptions^35–40^. Most studies on the effect of genetic alteration of complex I in mice have used mutation or deletion of the accessory subunit gene *Ndufs4* which is associated with phenotypes resembling human mitochondrial diseases such as Leigh syndrome and Leber Hereditary Optic Neuropathy^41,42^. Notably, loss of *Ndufs4* results in approximately 50% loss of mitochondrial complex I function^41^. In the present study, we sought to contribute to the understanding of the role of observed transcriptomic declines in mitochondrial complex I genes with advanced age by generating a mouse model that starts with a significant 50% reduction in mRNA for the gene encoding the core complex I subunit *Ndufs2* and characterizing the healthspan of these mice*.*

## Results

In mammalian cells, mitochondrial complex I, or NADH:ubiquinone oxidoreductase, is the largest complex in the ETC consisting of 45 individual protein subunits with a molecular weight approaching 1MDa^43,44^ (Fig. S1). There are 14 core subunits that are highly evolutionarily conserved from bacteria to eukaryotes that make up the physical core of the mature assembled mitochondrial protein complex. These core subunits house the key redox-active cofactors such as FMN, heme, and iron-sulfur clusters. Seven of the core subunits are encoded in the mitochondrial DNA (ND1, ND2, ND3, ND4, ND4L, ND5, ND6) and reside in the membrane arm of the assembled complex. The remaining 7 core subunits and the 31 supernumerary subunits are all encoded in the nucleus and must be imported into the mitochondria with the dedicated mitochondrial protein import machinery^45^.

NADH:ubiquinone oxidoreductase core subunit 2 (*Ndufs2*), encodes the 49 kDa core nuclear-encoded subunit which sits at an essential site at the interface of the membrane and matrix arms with an N-terminal loop in the membrane arm. *Ndufs2* makes up a significant part of the site of ubiquinone binding and is therefore essential for the enzymatic activity of mitochondrial complex I^46,47^, (Fig. S1). By contrast, NADH:ubiquinone oxidoreductase subunit S4 (*Ndufs4*) encodes an 18 kDa accessory subunit of complex I that does not directly participate in electron transfer. *Ndufs4* is instead thought to be an assembly factor that interacts with subunits of the matrix-facing N-module and the membrane associated Q-module of complex I which contributes to the stability of the fully assembled complex I^42^, (Fig. S1).

*Ndufs2* floxed mice have previously been described and *Ndufs2* has been implicated in organismal oxygen sensing^48^. *Ndufs2* deficiency has recently been shown to lead to a progressive Parkinson’s like phenotype when deletion is driven by a dopamine transporter cre recombinase^49^. *Ndufs2* deficiency specifically in neural progenitor cells in mice is associated with very early mortality by approximately 10 days of postnatal development^50^. Global deletion of *Ndufs2* is embryonic lethal while global loss of *Ndufs4* in most models is associated with viability for approximately 2 months of age^41^, however one point-mutant version of *Ndufs4* deficiency was also embryonic lethal^51^. Since *Ndufs4* mice have previously been described in more detail we sought to confirm the expected relative severity of *Ndufs2* to *Ndufs4* loss in the brain. In our previous study, we found that knockout of the accessory complex I subunit *Ndufs4* specifically in the brain using Nestin-cre can be complemented by expression of the yeast alternative NADH dehydrogenase NDI1 to increase lifespan^52^.

We crossed *Ndufs2* floxed mice to Nestin-Cre NDI1-LSL mice to generate brain specific *Ndufs2* deficient mice expressing the yeast NDI1. As expected, complete loss of *Ndufs2* specifically in the brain was associated with perinatal mortality (Fig. 2). Similar to our previous results with *Ndufs4* deficient mice, the yeast alternative NADH dehydrogenase was capable of increasing lifespan in mice with *Ndufs2* deficiency in the brain (Fig. S2). This result highlights the essential role of mitochondrial NADH oxidation activity in the brain for organismal survival, and the essential role of *Ndufs2* for complex I function^52^. These results also suggested to us that *Ndufs2* deficiency leads to relatively more profound reduction in complex I function as compared with *Ndufs4* deficiency.

**Figure 1:**
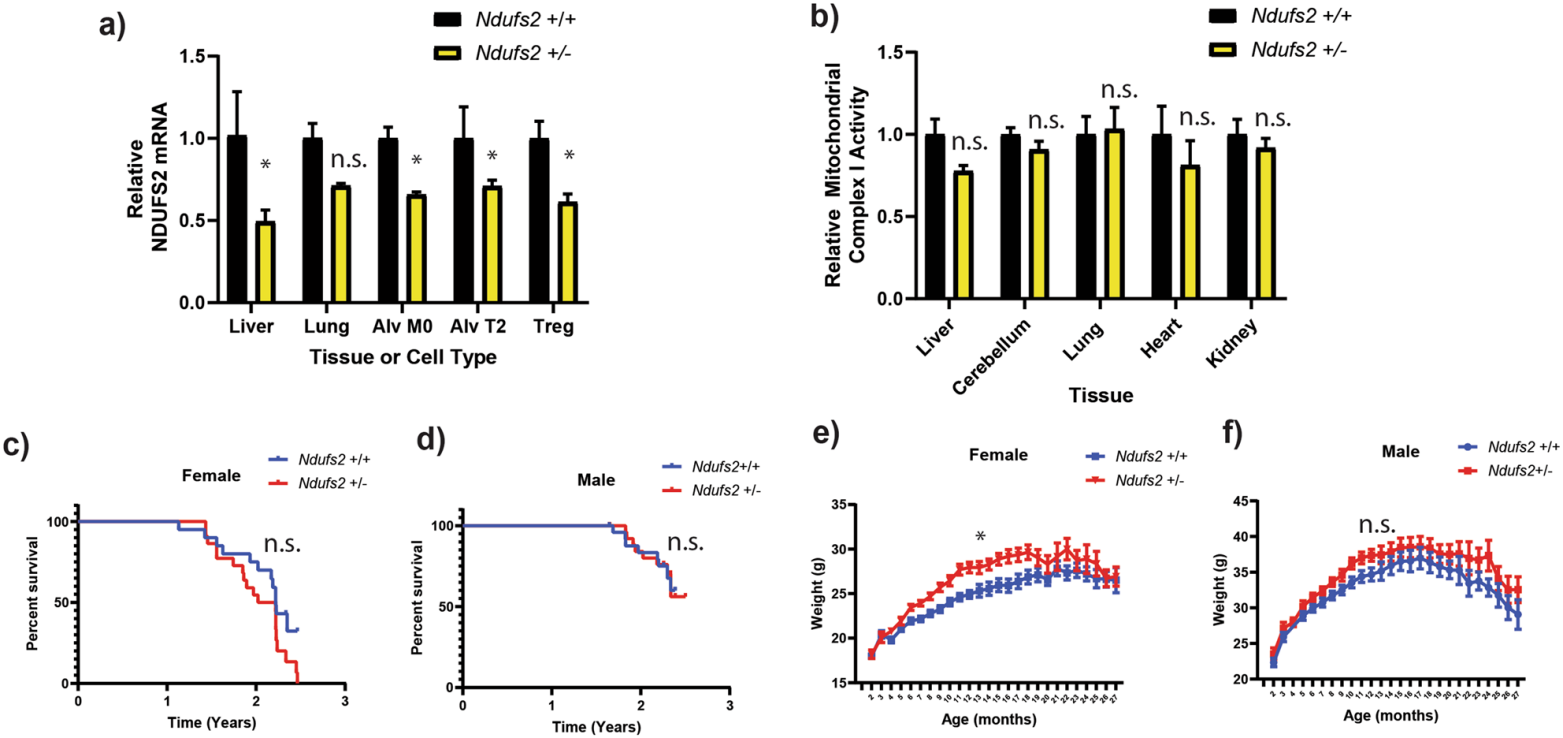
*Ndufs2* hemizygosity decreases mRNA but does not significant impair complex I function or lifespan. (**a**) *Ndufs2* mRNA quantification in cells and tissue. Two-way ANOVA column factor *p* < 0.0001, n = 2–4), Sidak’s multiple comparisons test *p < 0.05. (**b**) Relative complex I enzymatic activity in whole tissue. 2-way ANOVA column factor *p* = 0.0994 n = 5 Sidak’s multiple comparisons test not significant. (**c**) Survival of female *Ndufs2* ^+/+^ and *Ndufs2* ^+/−^ mice n = 20–22. Log-rank (Mantel-Cox) test *p* = 0.0791. (**d**) Survival of male *Ndufs2* ^+/+^ and *Ndufs2* ^+/-^ mice n = 28–29. Log-rank (Mantel-Cox) test *p* = 0.9842 (**e**) Average body weight by month of female *Ndufs2* ^+/+^ and *Ndufs2* ^+/−^ mice. Multiple t-tests adjusted *p < 0.01: Female *Ndufs2* ^+/−^ vs Female *Ndufs2* ^+/+^ months 6–12 and 16. N = 31–34. (**f**) Average body weight by month of male *Ndufs2* ^+/+^ and *Ndufs2* ^+/−^ mice. n = 22. n.s. not significant.

We next crossed *Ndufs2* floxed mice to Sox2-Cre ubiquitous expressing Cre mice to generate a global null allele of *Ndufs2.* The global null allele hemizygous *Ndufs2* mice were backcrossed to C57BL6/J WT mice and expanded for several generations to generate cohorts for aging. All cohorts were derived from the same backcross generation with > 95% C57BL6/J genetic identity based on SNP analysis. We measured *Ndufs2* mRNA in several tissues and sorted cell types, where we observed approximately a 50% reduction in the hemizygous *Ndufs2*^+*/-*^ mice (Fig. 1a). We extracted protein and measured total complex I NADH enzymatic activity from several tissues and observed similar levels between wild-type and hemizygous mice with statistically significant reduction in NDUFS2 protein observed in the brain but no significant or consistent trends towards lower protein levels or complex I activity across several tissues (Figs. 1b, S3a). We isolated cerebellar granule neurons from *Ndufs2*^+/-^ versus *Ndufs2*^+/+^ brains and measured basal oxygen consumption ex vivo. Basal oxygen consumption was similar in *Ndufs2*^+/−^ and wild-type neurons, suggesting hemizygous loss of *Ndufs2* is not limiting for ATP production (Fig. S3b). Together these data suggest that the 50% genetic reduction of the essential complex I subunit *Ndufs2* reduces mRNA without compromising mitochondrial ATP production, while complete loss of *Ndufs2* is lethal.

If modest levels of mitochondrial mRNA declines observed in aging are causally linked to shortening of lifespan or healthspan, we reasoned they should develop prematurely in aged *Ndufs2*^+/−^ mice. We did not observe any significant changes in early mortality between *Ndufs2*^+/+^ mice and *Ndufs2*^+/−^ male or female mice (Fig. 1c, d). We observed a trend towards a slightly higher body weight on average in both male and female *Ndufs2*^+/−^ mice compared to *Ndufs2*^+/+^ mice beginning at approximately 6 months of age, but the average body weights converged after 18 months of age (Fig. 1e, f). Histological analysis of multiple tissue types was carried out at greater than 24 months of age by a blinded mouse geropathologist, and no consistent differences were found between genotype groups in brain, heart, lung, liver, kidney, pancreas, spleen, colon, and quadriceps (Supplementary Table 1).

**Figure 2:**
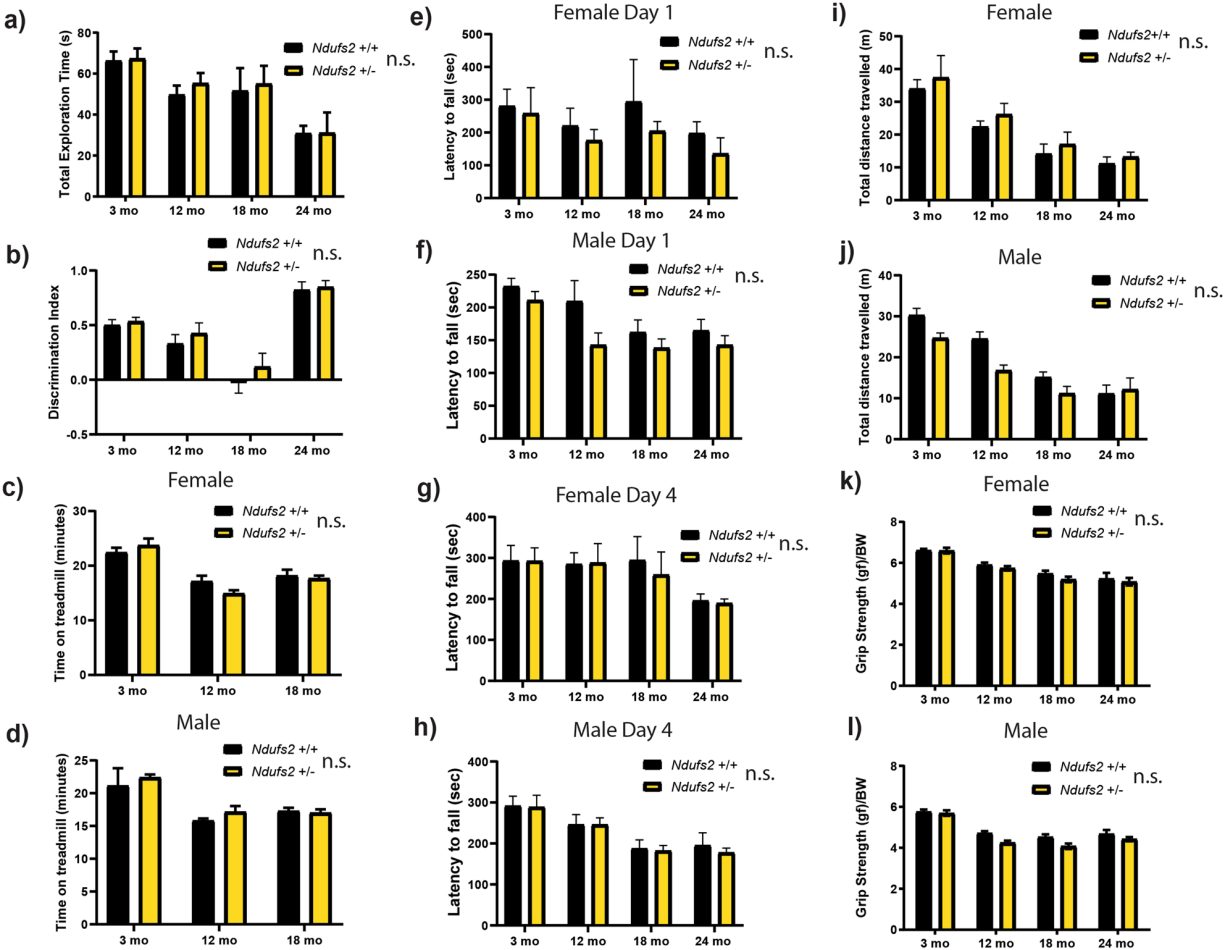
Longitudinal behavioral analysis of *Ndufs2*^+/-^ and *Ndufs2*^+/+^ mice. (**a**) Novel object recognition test total exploration time. Male and female data combined. n = 7–25 per group, multiple t-tests not significant. (**b**) Novel object recognition test discrimination index. Male and female data combined. n = 7–25 per group, multiple t-tests not significant. (**c**) Female forced maximal exercise on accelerating treadmill challenge total time on treadmill. n = 4–8. Multiple t-tests not significant. (**d**) Male forced maximal exercise on accelerating treadmill challenge total time on treadmill. n = 2–6. Multiple t-tests not significant. (**e**) Female day 1 accelerating rotarod challenge latency to fall. n = 4–13. Multiple t-tests not significant. (**f**) Male day 1 accelerating rotarod challenge latency to fall. n = 5–18. Multiple t-tests not significant. (**g**) Female day 4 accelerating rotarod challenge latency to fall. n = 4–13. Multiple t-tests not significant. (**h**) Male day 4 accelerating rotarod challenge latency to fall. n = 5–18. Multiple *t*-tests not significant. (**i**) Female open field test total distance. n = 4–13. Multiple *t*-tests not significant. (**j**) Male open field test total distance. n = 5–18. Multiple *t*-tests not significant. (**k**) Female grip strength. n = 4–13. Multiple *t*-tests not significant. (**l**) Male grip strength. n = 5–18. Multiple t-tests not significant. (**a**–**l**) Multiple t-tests refers to adjusted t-tests with a two-stage linear step-up procedure of Benjamini, Krieger and Yekutieli, with Q = 1% *Ndufs2*^+/+^ vs *Ndufs2*^+/−^ independently for each time point. n.s. not significant by genotype.

We measured several classic mouse behavioral tests in our cohorts of *Ndufs2*^+/+^ and *Ndufs2*^+/-^ mice at 3, 6, 12, 18, and 24 months of age, separated by sex to assess several aspects of motor and cognitive function. In a novel object recognition challenge there was a decreased overall exploration time with age but no difference between the genotypes (Fig. 2a). Discrimination index was not different between the genotypes (Fig. 2b). On a forced maximal exercise capacity treadmill challenge there were no consistent significant changes by genotype (Fig. 2c, d) with a trend towards decreased exercise capacity with age. On an accelerating rotarod challenge a trend towards a shorter latency to fall in *Ndufs2*^+/-^ male and female mice was observed on day 1 of the protocol (Fig. 2e, f). However, this trend was not present by the 4th day of the protocol which indicates intact motor learning (Fig. 2g, h). Performance on the rotarod and an open field challenge declined with age in all groups but there was no significant difference between the genotypes (Fig. 2e–j). Grip strength declined with age in all mice but was not significantly different between the genotypes (Fig. 2k, l). Declines in motor and cognitive function were observed with advancing age on all tests with all groups however there were not significant or consistent changes between *Ndufs2*^+/+^ and *Ndufs2*^+/−^ male or female mice in any of these behavioral tests across ages and sexes.

**Figure 3:**
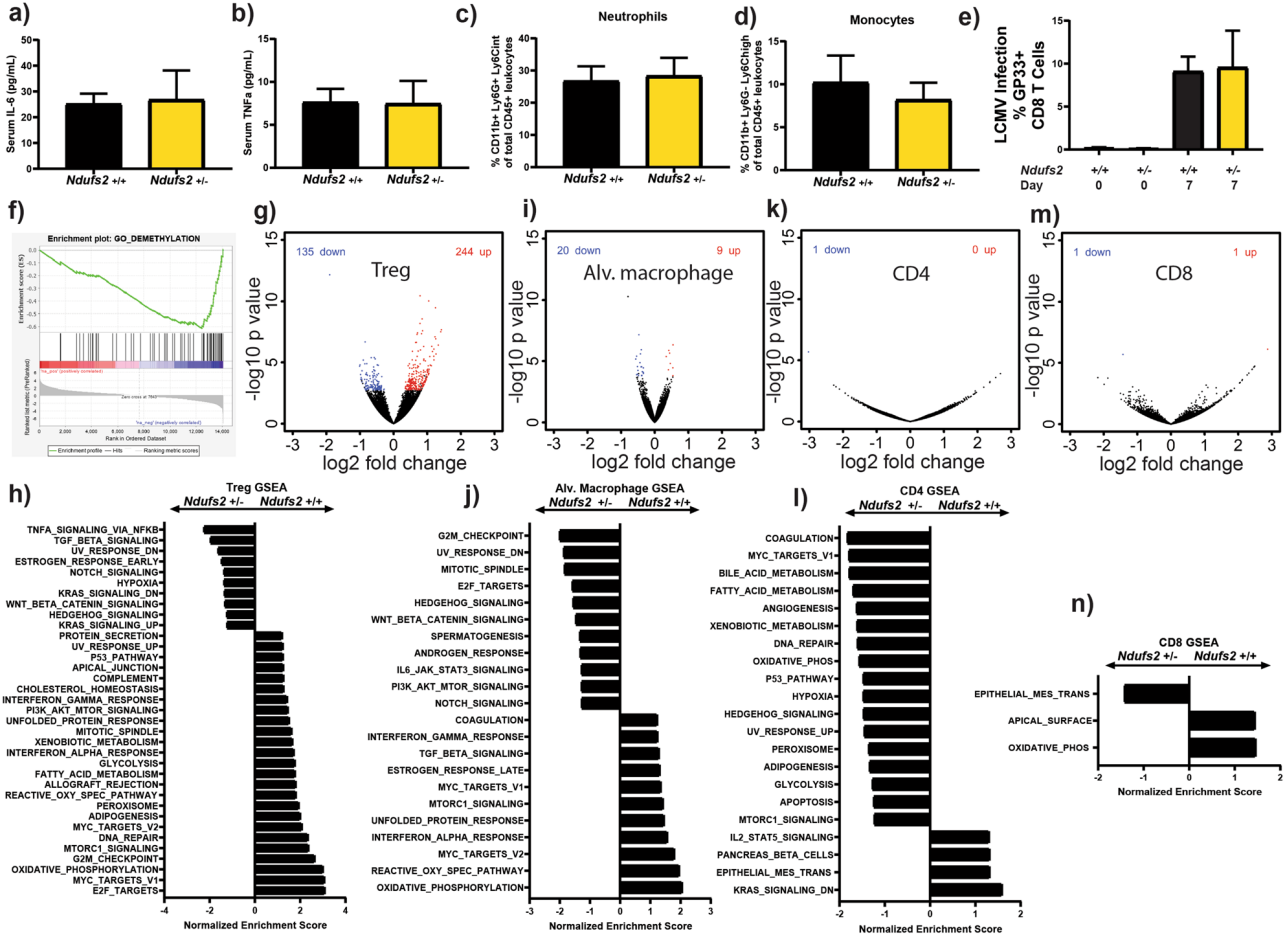
*Ndufs2* hemizygosity alters gene expression in immune cell populations. (**a**) Serum IL-6 measurement in 24 month-old mice. n = 4, unpaired t-test *p* = 0.8919. (**b**) Serum TNFa measurement in 24 month-old mice. n = 4, unpaired t-test *p* = 0.9616. (**c**) Leukocyte percentage of circulating neutrophils in 27-month-old mice. n = 4, unpaired *t*-test *p* = 0.8313. (**d**) Leukocyte percentage of circulating monocytes in 27-month-old mice. n = 4, unpaired *t*-test *p* = 0.6048. (**e**) Lymphocytic choriomeningitis virus (LCMV) infection response at 20 months of age. Percentage of GP33 responsive CD8 T cells at day 0 and day 7. n = 4, unpaired *t*-test *p* = 0.9204. (**f**) Regulatory T cell GSEA. Demethylation gene set is the top gene ontology biological process gene set enriched in *Ndufs2* ^+/-^ compared to *Ndufs2* ^+/+^ at 20 months of age. (**g**, **h**) Regulatory T cells differential gene expression analysis in 20-month-old mice. (**g**) Volcano plot individual gene FDR cutoff 0.05, n = 4 (**h**) GSEA significant MSigDB hallmark gene sets with FDR cutoff of 0.25, n = 4. (**i**, **j**) Alveolar macrophage differential gene expression analysis. (**i**) Volcano plot individual gene FDR cutoff 0.05, n = 4. (**j**) GSEA significant MSigDB hallmark gene sets with FDR cutoff of 0.25, n = 4. (**k**, **l**) CD4 cell differential gene expression analysis in 20-month-old mice. (**k**) Volcano plot individual gene FDR cutoff 0.05, n = 4 (**l**) Gene Set Enrichment Analysis (GSEA) Molecular Signatures Database (MSigDB) hallmark gene sets. Significant gene sets with FDR cutoff of 0.25, n = 4. (**m**, **n**) CD8 cell differential gene expression analysis in 20-month-old mice. (**m**) Volcano plot individual gene FDR cutoff 0.05, n = 4. (**n**) GSEA significant MSigDB hallmark gene sets with FDR cutoff of 0.25, n = 4.

Since mitochondrial function has emerged as a key regulator of immune cell function^53^ and dysregulation of immune function is a common feature in aging^54^, we sought to characterize the immune function of *Ndufs2*^+/-^ aged mice. Aging is characterized by increased circulating levels of IL-6 and TNFα^55^, and a shift in white blood cell percentages due to relative sparing of myeloid lineages and decreasing lymphocytes^56^. We measured circulating levels of the pro-inflammatory cytokines TNF-alpha and IL-6 and found no significant difference based on *Ndufs2* genotype in aged mice (Fig. 3a, b). There was no significant change in the percentage of circulating neutrophils (Fig. 3c) or monocytes (Fig. 3d) in aged *Ndufs2*^+/−^ vs *Ndufs2*^+/+^ mice. We next tested whether there were dramatic differences in an infection model between *Ndufs2*^+/+^ and *Ndufs2*^+/-^ mice. Therefore, we infected aged *Ndufs2*^+/+^ and *Ndufs2*^+/−^ mice with lymphocytic choriomeningitis virus (LCMV) and measured their response to infection by percentage of epitope specific CD8 T cells that bound to GP33 tetramer one week after infection. There were no significant differences between *Ndufs2*^+/+^ and *Ndufs2*^+/−^ in their response to this viral infection (Fig. 3e).

We next carried out transcriptomic analysis on several immunologic cell types isolated from aged mice (Fig. 3f–n). Notably, regulatory T cells (Tregs) demonstrated a few hundred significantly differentially expressed genes (Fig. 3g, S5a). Gene set enrichment analysis demonstrated hallmark gene set enrichment of oxidative phosphorylation, mTORC1, reactive oxygen species, glycolysis, and the unfolded protein response in *Ndufs2*^+/+^ Tregs relative to *Ndufs2*^+*/*−^ Tregs (Fig. 3h). Since Tregs are an important cell type in controlling inflammation, whose function are susceptible to metabolic changes, we carried out additional enrichment analyses using the Gene Ontology biological process gene sets and found that many of the top enriched pathways in *Ndufs2*^+/−^ Tregs involved methylation and demethylation (Fig. 3f). A network-based gene enrichment map generated from this analysis revealed a network of enriched gene sets involved in mitochondrial metabolism, RNA processing, DNA repair, cell division, and immunity in *Ndufs2*^+/+^ Tregs compared to *Ndufs2*^+/−^ (Fig. S4). There were fewer networks enriched in *Ndufs2*^+/−^ Tregs compared to *Ndufs2*^+/+^ with the notable exception of methylation and demethylation gene sets. Tregs are highly dependent on methylation/demethylation for maintaining their suppressive function, and methylation changes are commonly cited as a possible biomarker of aging^57,58^. We also directly visualized transcripts of the integrated stress response which has been identified as a set of genes that respond to several different mechanisms of induced mitochondrial stress^59,60^ . The lack of a clear pattern does not support the hypothesis that mitochondria in either Treg genotype are responding to an acute mitochondrial stress with age with the integrated stress response (Fig. S5b). While these transcriptomic changes are interesting and may suggest enrichment of genes associated with Treg function in the *Ndufs2*^+/+^ Tregs, the lack of an overt organismal inflammatory phenotype highlights that these transcriptomic changes may not reflect a true functional deficit in *Ndufs2*^+/-^ Tregs. There were fewer gene expression changes observed in alveolar macrophages (Fig. 3i, j), CD4 T cells (Fig. 3k, l) and CD8 T cells (Fig. 3m, n) with some overlap of enriched gene sets based on genotype. Together these results indicate that there may be complex cell and tissue type specific responses to falling levels of complex I gene transcripts during aging.

Since the antidiabetic drug metformin has been proposed as a potential pharmacological approach to target aging by a mechanism of complex I inhibition, we sought to analyze whether mild reduction in complex I mRNA in *Ndufs2*^+/−^ mice might potentiate or inhibit metformin’s effects. First, we sought to characterize steady state features of the important metabolic organs the kidney and the liver of *Ndufs2*^+*/*+^ vs *Ndufs2*^+*/*−^ mice at baseline and at advanced age with metabolomic and transcriptomic analyses (Figs. S6, S7). The metabolomic analysis of steady state metabolites did not reveal many significant changes based on age or genotype (Fig. S6a, b). Transcriptomic analyses of liver and kidney tissue did not demonstrate many significant changes at the level of individual genes between aged *Ndufs2*^+*/*+^ and aged *Ndufs2*^+*/*−^ mice (Fig. S7a, b). Gene set enrichment analysis revealed hallmark gene sets enriched in aged *Ndufs2*^+/−^ including oxidative phosphorylation, fatty acid metabolism, MYC targets and heme metabolism. Gene sets enriched in *Ndufs2*^+/+^ liver included cholesterol homeostasis, TNFα, IL2/STAT5, and IL6/JAK/STAT3 signaling pathways (Fig. S7c). There were several genes that were significantly dysregulated among groups, but unsupervised clustering of the groups segregated more by age than genotype (Fig. S7d). Hallmark gene sets enriched in aged *Ndufs2*^+/−^ kidney also included oxidative phosphorylation, fatty acid metabolism, and MYC targets. However, in kidney additional gene sets enriched in *Ndufs2*^+/−^ tissues included IL6/JAK/STAT3, unfolded protein response, inflammatory response, and reactive oxygen species pathway (Fig. S7e). Using a similar ANOVA like test on individual genes and unsupervised clustering, we again saw that groups segregated more by age than by genotype (Fig. S7f).

**Figure 4:**
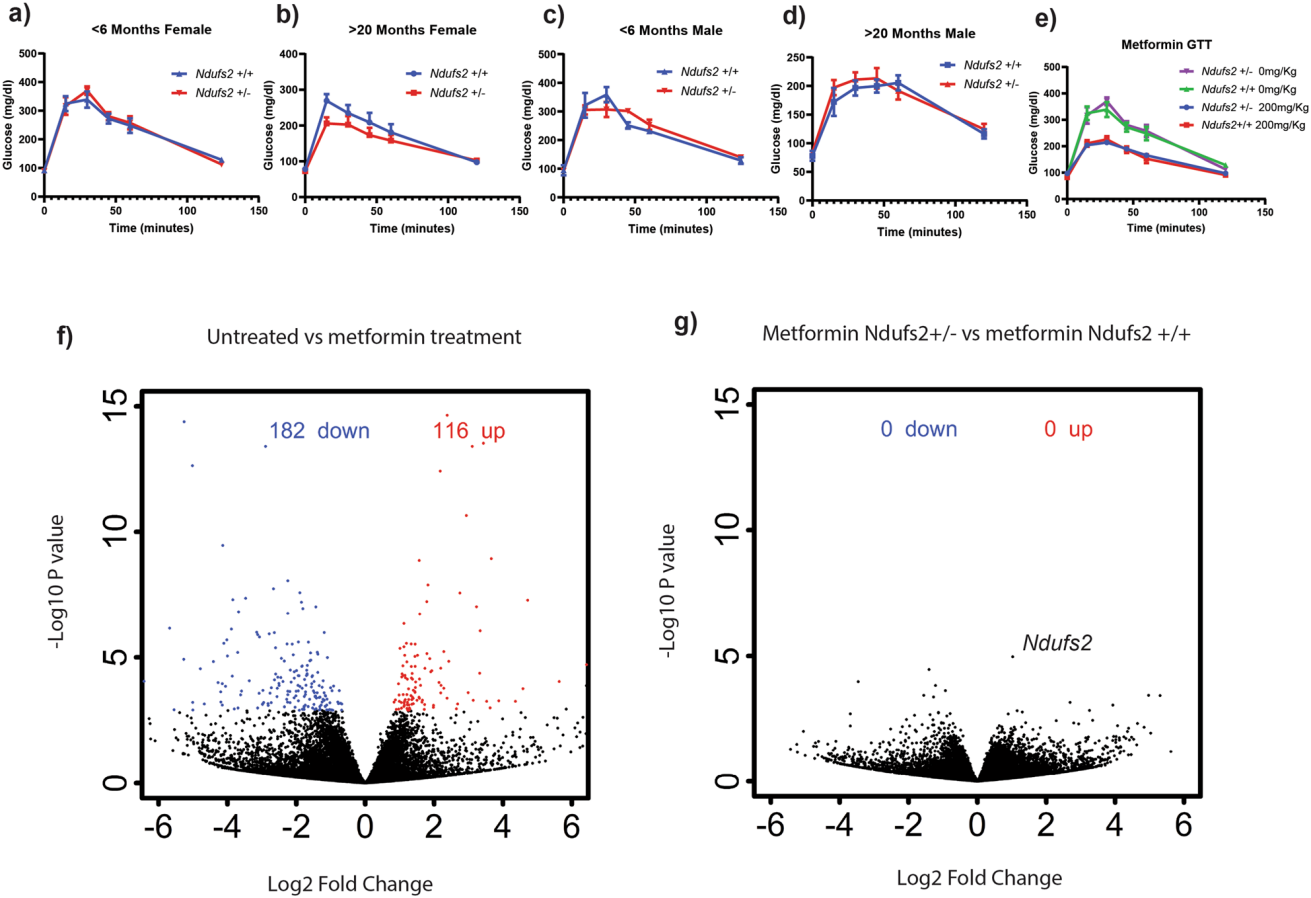
*Ndufs2* hemizygosity minimally impacts organismal responses to acute metformin treatment. (**a**–**d**) Multiple t-tests, one per time point *Ndufs2*^+*/*+^ vs *NDUFS2*^+*/*−^ discovery determined using the Two-stage linear step-up procedure of Benjamini, Krieger and Yekutieli, with Q = 0.05%. Each row was analyzed individually, without assuming a consistent SD. No discoveries. Error bars represent standard error. (**a**) Glucose tolerance test for females < 6 months of age n = 4–6. (**b**) Glucose tolerance test females > 20 months of age n = 7–11 per group (**c**) Glucose tolerance test for males < 6 months of age n = 2–3 per group. (**d**) Glucose tolerance tests for males > 20 months of age n = 5–7. (**e**) Oral glucose tolerance test (GTT) with or without 30-min pre-treatment with 200 mg/Kg metformin in *Ndufs2*^+/+^ and *Ndufs2*^+/−^ mice. Ordinary one-way ANOVA *p* = 0.4275, Sidak’s multiple comparisons tests by genotype not significant. n = 4–8. Error bars represent standard error. (**f**) Liver tissue differential gene expression analysis from untreated *Ndufs2*^+*/*+^ mice versus *Ndufs2*^+*/*−^ mice 8 h after treatment with 200 mg/Kg metformin. Volcano plot individual gene FDR cutoff 0.05. n = 3,5. (**g**) Liver tissue differential gene expression analysis after 8 h of treatment with 200 mg/Kg metformin in *Ndufs2*^+*/*+^ vs. *Ndufs2*^+/−^ mice. Volcano plot individual gene FDR cutoff 0.05. n = 5.

Metformin acutely alters circulating glucose levels after oral glucose intake. Therefore, glucose tolerance testing was used to determine whether *Ndufs2* genotype influenced whole body glucose disposal. While no significant changes were observed in the younger time points, aged females had a trend towards a slight decrease in peak glucose concentrations in female *Ndufs2*^+/−^ mice which could point towards more rapid glucose disposal, however this was not observed in male *Ndufs2*^+/−^ mice (Fig. 4a–d). Next, we measured the acute effect of metformin on glucose disposal using a glucose tolerance test after pretreatment with an oral bolus of 200 mg/Kg metformin. Metformin reduced the peak blood glucose levels in both *Ndufs2*^+/−^ and *Ndufs2*^+/+^ mice to similar levels (Fig. 4e). Next, we measured the metformin-responsive mitochondrial stress hormone GDF15 acutely upon treatment with metformin. At 4 and 8 h there was a measurable increase in circulating levels of GDF15 that was not significantly different based on genotype (Fig. S8a). We next carried out transcriptomic analysis of liver and kidney tissue with and without metformin treatment (Figs. 4a, b, S8b, c, S9). Acute metformin treatment results in several hundred gene expression changes in the liver when compared to untreated liver (Fig. 4f). However, comparing metformin-treated *Ndufs2*^+*/*+^ liver gene expression to metformin-treated *Ndufs2*^+*/*−^ liver gene expression revealed no changes at the level of individual genes (Fig. 4g). Consistent with the trend in serum GDF15 levels, *Gdf15* transcript was significantly induced in liver tissue by treatment with metformin. There no significant pairwise difference by genotype and no increase in aged mice (Fig. S8b). Similarly, levels of hepatic *Fgf21* mRNA, which like *Gdf15* responds to mitochondrial stress and metformin treatment, were increased with acute metformin treatment. There was no difference by genotype and again there was no increase in aged mice (Fig. S8c). Acute treatment with metformin resulted in numerous statistically significant gene expression changes in kidney and liver (Fig. S9c, f). Unsupervised clustering of significant genes reveals that the majority of this change can be explained by treatment rather than genotype (Fig. S9c, f). Gene set enrichment analysis of treated versus untreated tissue demonstrated numerous significantly enriched pathways with significant overlap among groups (Fig. S9a–e). Metformin treatment consistently led to an enrichment of oxidative phosphorylation, MYC targets, reactive oxygen species pathway, fatty acid metabolism, DNA repair, and apoptosis gene sets in both *Ndufs2*^+/+^ and *Ndufs2*^+/−^ liver and kidney tissues after 8 h of treatment. Taken together, these results indicate that *Ndufs2*^+/−^ and *Ndufs2*^+/+^ respond similarly to treatment with metformin.

## Discussion

Overall, our data indicate that our attempt to mildly inhibit mitochondrial complex I activity by genetic loss of one allele of *Ndufs2* in mice did not result in significant benefits or detriments for lifespan or healthspan. Our oxygen consumption and complex I enzymatic activity data and scarcity of metabolite changes in the highly metabolic liver and kidney tissues do not suggest a significant impairment of mitochondrial complex I function. The reduced *Ndufs2* mRNA in this model was not sufficient to induce lifespan or healthspan effects with aging, and any slight differences in this model might require a more aggressive metabolic or immunologic challenge to uncover. Our results suggest that modest to moderate declines in expression levels of mitochondrial genes, in the absence of evidence of a decline of mitochondrial function, should be interpreted with caution as we have observed few phenotypic consequences in aging mice with a 50% reduction in mRNA for a core catalytic subunit of complex I. These results may also be consistent with a recent bioinformatic study suggesting that mitochondrial genes are not under strong purifying selective pressure as compared with nuclear-related and transcription factor encoding genes in the context of heritable susceptibility to numerous aging related pathologies^61^.

There is an extensive literature on mice with deletion and mutation of the complex I subunit *Ndufs4* which is a model used primarily for studying primary mitochondrial disease^42^. There are several different versions of the *Ndufs4* deficient mouse that have been reported, although the majority make use of an exon-2 floxed allele for tissue specific deletion or a null allele that was generated with deletion of exon two resulting in a frame shift^41^. In addition to the exon 2 deleted *Ndufs4* mice, additional *Ndfus4* alleles reported include a knock-in point mutant allele^51^, a B2 SINE-inserted *fky* allele^62^, and an exon 1 gene trap allele^63^. Knockout of both alleles of *Ndufs4* in the most widely used exon 2 model leads to early mortality at approximately 2 months of age accompanied by a necrotizing encephalopathy that resembles human Leigh syndrome and display approximately 50% complex I activity^41,64^. By contrast, the gene trap exon 1 allele homozygous mice display a slightly more mild phenotype with approximately a 25% reduction in complex I activity, the B2 SINE-inserted *fky* homozygous mice demonstrated a more severe phenotype with earlier onset of disease and greater than a 70% reduction in complex I activity, and the knock-in point mutant allele homozygous mice were found to be embryonic lethal^51,62,65^. Hemizygous exon 2 *Ndufs4* mice were originally reported to be phenotypically normal without a significant decrease in complex I function^41^, but follow up research has suggested that there is a small but detectable decrease in complex I levels and increased sensitivity to myocardial reperfusion injury^66^. Mice heterozygous for the *fky* allele were found to be phenotypically normal and did not have a reported decrease in complex I activity^62^. Mice heterozygous for the knock-in *Ndufs4* point mutation, however, did demonstrate an approximate 25% reduction in complex I activity in several tissues unlike the other *Ndufs4* heterozygote models^51^. Comparing the phenotype of the most widely used *Ndufs4* homozygous knockout to the mice in our study clearly demonstrates that there is a large difference. *Ndufs4* deficient mice have low body weight, neurological dysfunction, and early mortality, none of which is seen in the present study, which clearly demonstrates that the *Ndufs2* hemizygous mice are not a reasonable model of mitochondrial disease^41^.

In addition to the *Ndufs4* mouse models, several other studies with different genes related to the ETC have been carried out. Homoplasmic mutant mice for the mitochondrially encoded complex I subunit *mt-Nd2* have a mildly shortened lifespan and are susceptible to glucose intolerance^35^. A study in WT mice found that mice that would go on to live longer had a reduction in the matrix subunits of complex I at a young age^36^. Hemizygosity of the mitochondrial complex IV (COX) subunit SURF1 does not change healthspan despite COX activity being reduced in tissue (22–87%)^37^. In mice with skeletal muscle-specific knockout of COX10, COX activity was greater than 95% reduced compared to control but muscle function was minimally impacted at baseline (0–20%)^38^. Hemizygosity for the essential ubiquinone biosynthetic enzyme *Mclk1* demonstrates increased lifespan while increasing markers of mitochondrial oxidative stress and decreasing mitochondrial oxidative phosphorylation^39^. Hemizygosity of the essential complex III subunit *Uqcrfs1* (RISP) also did not impact lifespan overall, but did show a mild decrease in lifespan in males but not females^40^.

We would speculate that our results are most comparable to the widely used *Ndufs4* hemizygous mice, which show a normal body weight, normal longevity, and a relative lack of phenotype^42^. We did not see a statistically significant decrease in complex I activity in our mice. Furthermore in most tissues the capacity for ATP production far exceeds basal rates of ATP consumption which are primarily determined by the activity of cellular ATPases^67^. Mitochondrial electron transport usually only becomes limiting with reductions in mitochondrial electron transport that exceed 70% or during transient periods of extreme metabolic demand, for example rapidly contracting skeletal muscle^68–71^. While the exact correlation of electron transport chain (ETC) enzymatic activity and ATP production is likely dependent on the tissue and metabolic context, in most studies of aging and aging associated disease, investigators report a 30–50% reduction in mitochondrial enzymes or function, far from the 70% threshold^13,72^. Our model in the context of the *Ndufs4* literature may reflect that the amount of mRNA for both *Ndufs4* and *Ndufs2* is typically in excess of what is required for normal complex I activity and is therefore unlikely to be rate-limiting with normal aging.

There are gene expression changes in several cell types caused by loss of one allele of *Ndufs2.* This indicates that there may be a subtle cellular response to the 50% reduction in this essential complex I component transcript. Recently, the master regulator of mtDNA, TFAM, was deleted in T cells leading to mitochondrial dysfunction and resulted in a premature aging phenotype in mice^73^. Complete loss of TFAM is very different than our model, and results in dramatic reduction in mtDNA as well as ETC components leading to profound mitochondrial dysfunction that is distinct from the loss of single ETC components alone. Complete loss of ETC component RISP only in Tregs results in dramatic whole-body inflammation and a short lifespan^74^. A loss of Treg suppressive activity with advanced age has been postulated to underlie the observed sterile inflammation that occurs with aging in addition to metabolic dysregulation of other T cell subsets ^73,75^. Our data on the differential gene set enrichment in aged immune cells without a clear difference in infection susceptibility is potentially interesting in the context of recent work on the intersection of aging and influenza susceptibility which has demonstrated that mitochondrial protein levels are variably impacted in different cell populations with both aging and viral infection^76^. Taken together these studies suggest that mitochondrial functional changes with aging may lead to highly cell-type dependent pathophysiological mechanisms of disease susceptibility rather than a single unifying mechanism to explain how mitochondrial function in all cells are impacted by, and influence, the diverse sequela of aging.

The widely used antidiabetic drug metformin has been identified as a possible anti-aging medication that may mediate its preventative effects on several aging-related pathologies through mild inhibition of mitochondrial complex I^77,78^. Our results indicate that *Ndufs2*^+/−^ mice are still able to respond normally to acute treatment with metformin. These results are intriguing in the context of the current interest in the use of metformin and its role in mitochondrial function, as an agent for the primary prevention of aging-related pathology^79^, but are not conclusive in the setting of intact complex I function. Our data provide some support to the idea that declines in mitochondrial ETC component mRNA that are frequently observed and reported in aging transcriptomic studies may be correlative rather than causal in aging-related pathology.

**Figure 5:**
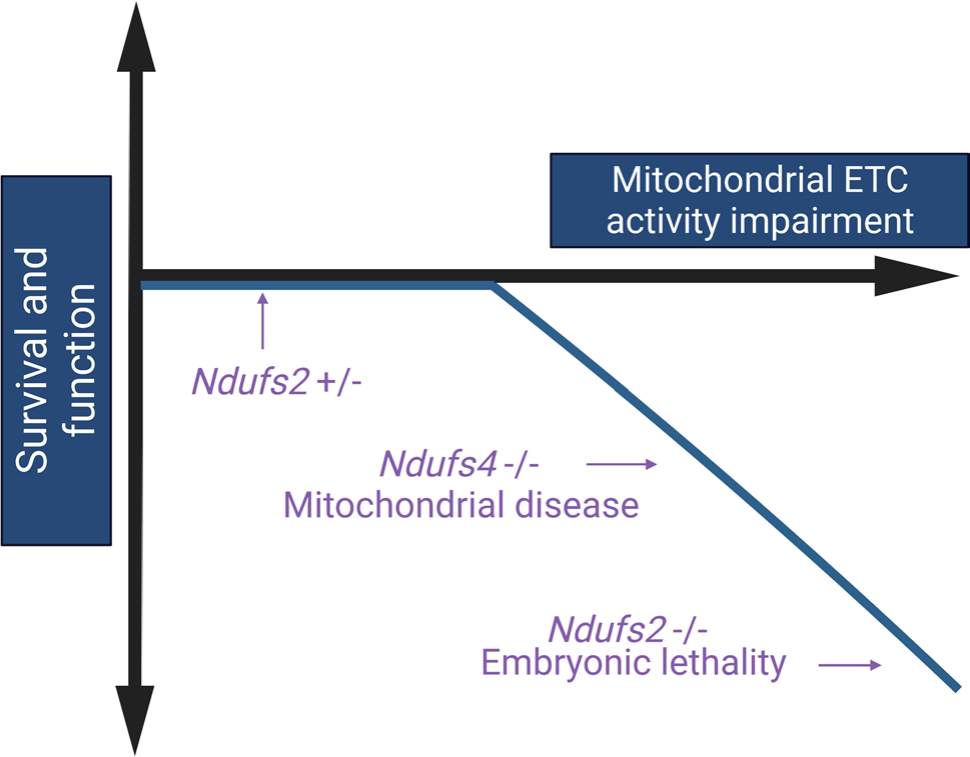
Proposed qualitative model of the relationship between mitochondrial electron transport chain impairment and survival and function in vertebrates. Created with https://biorender.com.

The exact relationship between the degree of electron transport chain activity impairment and organismal survival and function remains a challenge to understand. Based on the paradoxical invertebrate results of ETC knockdown in both life extension and early mortality, this relationship between survival/function and mitochondrial activity impairment can be theoretically formulated as biphasic hormesis response (Fig. 5). Clearly there is an association of normal aging with a degree of ETC impairment, but this impairment is not as severe as that seen in models of primary mitochondrial diseases or in embryonic lethal complete knockouts of essential ETC components like *Ndufs2.* Interventions like caloric restriction, exercise, and metformin treatment have all been associated with increased life or healthspan as well as a mild degree of mitochondrial ETC activity impairment that is thought to trigger a beneficial stress response. We would speculate that our intervention in this study, which was a reduction in *Ndufs2* mRNA, was too mild to rise to the level of measurable ETC impairment and was therefore insufficient to induce either a beneficial response or a harmful response (Fig. 5). A major but challenging goal of the aging field should be to determine definitively if interventions that improve or impair survival and function at the broad level of organismal health require mitochondrial dysfunction, such that causality of this relationship can be understood with more precision.

## Methods

### Mouse models

We generated *Ndufs2* hemizygous mice by crossing *Ndufs2* floxed^48^ and B6.Cg-Edil3 Tg(Sox2-cre)1Amc/J Sox2-Cre mice (Jax Strain 008454). We sent samples from the *Ndufs2* floxed mice that we used to generate *Ndufs2* global hemizygote to Jackson labs for SNP analysis. This came back as approximately 95% Bl6/J and 5% 129S1/SvImJ. The SOX2 cre mice from Jackson that we used to generate the *Ndufs2* global background are on a mixed C57BL6J/N background. The SOX2 cre mice were backcrossed for 3 generations to C57BL6/J in house prior to breeding with the *Ndufs2* floxed mice. Then following the generation of the global heterozygous mice there were 3 more backcross generations to C57BL6/J to generate the mice in the cohort. Nestin cre (Jax strain 003771) and NDI1-LSL mice were previously described^52^. Experimenters were blinded to genotype during all described experiments. Mice were housed at Northwestern University Center for Comparative Medicine under specific pathogen-free conditions in accordance with federal and university guidelines for the care and use of experimental vertebrate animals and protocols approved by the Northwestern Institutional Animal Care and Use Committee (IACUC). All animal experiments and procedures were reviewed and approved by the IACUC at Northwestern University and were performed in accordance with federal and university guidelines and regulations for the care and use of experimental vertebrate animals. Authors have complied with the ARRIVE guidelines for reporting.

- *Ndufs2* genotyping primers:
  - Forward primer: 5′-ATAAGAGTGGATAGGATGTTT-3′.
  - Flox reverse primer: 5′-CATTTCTCCCTTCCCGTC-3′.
  - Null allele reverse primer: 5′-AGTGGCAGAACAATAGAGTGATCCAGGG-3′.
- Cre genotyping primers (Jax labs strain # 008454) Sox2 cre.
  - Cre Forward: 5′-GCAGAACCTGAAGATGTTCGCCAT-3′
  - Cre Reverse: 5′-AGGTATCTCTGACCAGAGTCATCC-3′
  - Internal Control Forward: 5′-CTAGGCCACAGAATTGAAAGATCT-3′
  - Internal Control Reverse: 5′-GTAGGTGGAAATTCTAGCATCATCC-3′

### Protein and mRNA quantification

Protein quantification was carried out on protein extracts from mouse tissues as previously described^52,80^. Mice were euthanized and tissue was flash frozen in liquid nitrogen. Protein was extracted using lysis buffer (Cell Signaling 9803S) and rotor–stator homogenization (QIAGEN TissueRuptor II). Protein was quantified using the Pierce BCA assay (Thermo Fisher Scientific) and NDUFS2 and Vinculin were measured using a ProteinSimple Wes Simple Western System, an automated capillary based immunoassay. Approximately 50ug of total protein was loaded for each sample. Antibodies used were: anti-*Ndufs2* (Abcam; Catalogue Number: ab96160; used at a 1:100 dilution), anti-Vinculin (Cell Signaling; catalog number: 13901 s, used at a 1:1000 dilution).

RNA was extracted using a QIAGEN RNeasy Plus Micro Kit following manufacturer protocol and qPCR was carried out using a CYBRFast 1-step RT-qPCR Lo-Rox Kit (Tonbo Biosciences) following manufacturer protocol with a BioRad CFX384 Real Time System. n = 2–4 biological replicates per group as noted in figure legends.

*Ndufs2* qPCR Primers:

- 5′-TTTCGGGAGCTGTCATGTACC-3′
- 5′-TGGTCACCGCTTTTTCCTTCA-3′

#### Glucose tolerance testing

Mice were fasted overnight. A small incision was made at the tail base for blood glucose measurements, and fasting glucose was measured using a glucometer. Mice were given a glucose bolus by oral gavage at a dose of 2 g per kilogram of body weight from a 0.2 mg/µL stock solution of glucose in sterile water. Glucose measurements were recorded at 15 min, 30 min, 45 min, 60 min, and 120 min after glucose bolus delivery. n = 2–11 biological replicates per group as noted in figure legends.

### RNA sequencing

Transcriptomics were carried at as previously described^52,80^. Mice were euthanized. Lungs were perfused with PBS and then instilled with Dispase and DNase, minced, and digested, then filtered, and centrifuged, red blood cells were lysed, and cells were stained for sorting. Alveolar macrophages were isolated as previously described^78^. Splenic T-regs were isolated using a Stemcell Technologies EasySep Mouse CD4 + CD25 + Regulatory T Cell Isolation Kit II following manufacturer protocols. CD4 and CD8 T cells were simultaneously cell sorted by FACS RBC-lysed whole blood. Liver and kidney tissue were dissected and flash frozen and disrupted during RNA isolation using a Qiagen TissueRuptor. RNA was extracted using Qiagen All-Prep DNA/RNA extraction kits following manufacturer protocols. RNA was quantified and QC was checked using an Agilent 4200 TapeStation RNA ScreenTape. mRNA libraries were prepared using NEBNext Ultra Kit with polyA selection (New England BioLabs). Libraries were sequenced using a Next-Seq 500 High Output for 75 cycles (Illumina). Raw BCL read files were demultiplexed and FASTQ files were generated using bcl2fastq and trimmed using Trimmomatic. Next, the reads were aligned to the mouse mm10 reference genome using STAR to generate BAM files. HTSeq was used to count reads in the exons of genes. Pairwise differential gene expression analyses were carried out using the R package DESeq2. Gene set enrichment analysis was carried out using the Broad Institute GSEA software after conversion to human homologue gene symbols. The pairwise differential gene expression output generated from DESeq2 was submitted as a pre-ranked list based on the DESeq2 Wald Statistic (stat column) output sorting genes from significantly upregulated to significantly downregulated. GSEA was performed on Hallmark Gene Sets from the GSEA Molecular Signatures Database or Gene Ontology Biological Process gene sets. Gorilla (Gene Ontology enrichment analysis and visualization tool) was used for additional gene ontology analysis. n = 3–6 biological replicates as noted in figure legends.

### Gene network analysis

Gene Set Enrichment Analysis software and Cytoscape software were used to generate gene network analysis plots. Preranked lists were analyzed for gene set enrichment for all 7350 Gene Ontology (GO) Biological Process gene sets on the Molecular Signatures Database. Next, GSEA results were loaded into the Enrichment Map Visualization tool in GSEA and Cytoscape software with enrichment map parameters set to a *p*-value cutoff of 0.005 and an FDR Q-value cutoff of 0.05 a similarity cutoff using an overlap coefficient of 0.5. n = 4 biological replicates per group.

### Metabolite measurements

Metabolomics were carried out as previously described^52,80^. Mice were euthanized and tissues were rapidly isolated, and flash frozen in liquid nitrogen. Samples were stored at − 80 °C until extraction. Soluble metabolites were extracted directly from tissue using cold methanol/water (80/20, v/v) at approximately 1 µL per 50 µg of tissue. Tissue was disrupted for 15 s by rotor–stator homogenization (QIAGEN TissueRuptor II). Protein was precipitated by incubation at − 80 °C. Debris were pelleted by centrifugation at 18,000xg for 15 min at 4 °C. The supernatant was transferred to a new tube and evaporated to dryness using a SpeedVac concentrator (Thermo Savant). Metabolites were reconstituted in 50% acetonitrile in analytical-grade water, vortex-mixed, and centrifuged to remove debris. Samples were analyzed by Ultra-High-Performance Liquid Chromatography and High-Resolution Mass Spectrometry and Tandem Mass Spectrometry (UHPLC-MS/MS). Specifically, the system consisted of a Thermo Q-Exactive in line with an electrospray source and an Ultimate3000 (Thermo) series HPLC consisting of a binary pump, degasser, and auto-sampler outfitted with an Xbridge Amide column (Waters; dimensions of 4.6 × 100 mm and a 3.5 μm particle size). Mobile phase A contained 95% (vol/vol) water, 5% (vol/vol) acetonitrile, 10 mM ammonium hydroxide, 10 mM ammonium acetate, pH = 9.0; and mobile phase B was 100% Acetonitrile. The gradient was as follows: 0 min, 15% A; 2.5 min, 30% A; 7 min, 43% A; 16 min, 62% A; 16.1–18 min, 75% A; 18–25 min, 15% A with a flow rate of 400μL/min. The capillary of the ESI source was set to 275 °C, with sheath gas at 45 arbitrary units, auxiliary gas at 5 arbitrary units, and the spray voltage at 4.0 kV. In positive/negative polarity switching mode, an m/z scan range from 70 to 850 was chosen and MS1 data was collected at a resolution of 70,000. The automatic gain control (AGC) target was set at 1 × 10^6^ and the maximum injection time was 200 ms. The top 5 precursor ions were subsequently fragmented, in a data-dependent manner, using the higher energy collisional dissociation (HCD) cell set to 30% normalized collision energy in MS2 at a resolution power of 17,500. Data acquisition and analysis were carried out by Xcalibur 4.1 software and Tracefinder 4.1 software, respectively (both from Thermo Fisher Scientific). The peak area for each detected metabolite was normalized by the total ion current which was determined by integration of all of the recorded peaks within the acquisition window. Downstream analysis was carried out using Microsoft Excel and R. Statistical analysis using multiple comparisons adjusted T-tests, and plot generation were performed using MetaboAnalyst 4.0 (Chong et al., 2018). n = 3–4 biological replicates per group.

### Complex I activity assays

Colorimetric Complex I Enzyme Activity Microplate Assay Kits (Abcam ab109721) were used to analyze complex I activity in post-mortem mouse tissues using manufacturer’s instructions. n = 5 biological replicates per group.

### Histological analysis and scoring

Mice were euthanized and tissues were fixed in 4% paraformaldehyde. Fixed tissues were paraffin embedded, sectioned, and hematoxylin and eosin stained at the Northwestern University Mouse Histology and Phenotyping Laboratory. Histological analysis and scoring was performed blinded at the Geropathology Research Network at the University of Washington. n = 5–7 biological replicates per group.

### ELISA (IL6, TNFa, GDF15)

Circulating levels of IL6, TNFa, and GDF15 were analyzed in mouse serum using R&D Systems ELISA kits (DY406, DY410, MGD150) following manufacturer’s protocols. n = 4–6 biological replicates per group.

#### Metformin treatment

Mice were treated with 100 or 200 mg/Kg metformin in water by acute oral gavage either 30 min prior to glucose tolerance testing or for 8 h to assess acute tissue transcriptional changes. Metformin was obtained from Sigma (PHR1084-500MG). n = 4–6 biological replicates per group as noted in the figure legends.

#### LCMV infection model

Mice were infected with lymphocytic choriomeningitis virus (LCMV) by intraperitoneal injection at 2 × 10^5^ PFU. Blood was collected retro-orbitally, red blood cells were lysed, leukocytes were antibody-stained and analyzed by flow cytometry. n = 4 biological replicates per group.

#### Flow cytometry

Blood was collected from mice and red blood cells were lysed. An antibody panel for a white blood cell differential was used to stain leukocytes, which were subsequently analyzed on a BD FACS Symphony at the Northwestern Flow Cytometry Core Facility. Antibody panels shown below:

White Blood Cell Differential:

**Table:** 

Laser	Laser	Filter	Dye	Antigen	Clone	Vendor
Violet	405	450/50	eFluor450	CD11b	M1/70	Biolegend
		525/50	V500	CD45	30-F11	BD
Green	552	575/25	PE	CD115	AFS98	BD
		780/60	PECy7	CD62L	MEL-14	eBio
Red	640	670/30	APC	CD43	S11	Biolegend
		780/60	ACPCy7	Ly6C	HK1.4	Biolegend
Far Red	690	730/45	Alexa 700	Ly6G	18A	BD

LCMV Panels:

Day 0:

APC-gp33 tetramer

Pecy7-CD8a.

APCef780-CD45.2

BV785-CD44

Day 7+:

APC-gp33 tetramer

Pecy7-CD8a

PerCPcy5.5-CD62L.

BV785- CD44

### Open field behavioral test

Mice were tested as previously described^81,82^. On the day of testing, mice were acclimated in the testing room 30 min prior to testing. To test, mice were placed in the open arena (acrylic box: 44 × 44 × 44 cm) and allowed to explore the area in the box for 10 min. Activity in the open field was recorded by digital video camera and Any-maze software (Stoelting Inc.) was used to score behavior in open-field testing. Parameters analyzed include total distance travelled, mean speed, time spent in the central and peripheral zones and duration of the immobility (resting time in seconds) in the arena. Statistical analysis was performed using one-way ANOVA (followed by Tukey’s honest significant difference post-hoc test for multiple comparisons), using GraphPad Prism 7 software (GraphPad Software, La Jolla California USA). Data were considered significant for *p* < 0.05. Data are expressed as mean ± SEM. n = 4–18 biological replicates per group.

### Rotarod behavioral test

Mice were tested as previously described^81,82^. Mice were placed on a rotating rod apparatus (Ugo Basile) that accelerates linearly from 4 to 40 rotations per minute over a 5 min period. Mice were subjected to 4 trials per day for 4 consecutive days, each trial lasting to a maximum of ten minutes, with at least ten minutes of rest between each trial. The average performances for each day were plotted, and statistical differences between the different groups were analyzed using repeated measures two-way ANOVA (followed by Tukey’s honest significant difference post-hoc test for multiple comparisons). All statistical analyses here and elsewhere were performed using GraphPad Prism 7 software (GraphPad Software, La Jolla California USA). Data were considered significant for *p* < 0.05. Data are expressed as mean ± SEM. N = 4–18 biological replicates per group.

### Grip strength behavioral test

Mice were tested as previously described^81,82^.Forelimb skeletal muscle strength was assessed using a digital grip strength meter (Columbus Instruments, Columbus, OH). The mice were picked up by the tail from the cage and then allowed to rest on a hard surface with all four paws on the flat surface. Then the mouse was lifted by its tail and was gently and slowly lowered over the top of the horizontal mesh grid attached to the grip meter, such that only its front paws can grip the grid. The mouse was allowed to grasp the mesh grid with its forelimbs and will then be gradually pulled backward steadily along a horizontal plane until the grid is released. When the animal releases the grid, the maximal grip strength will be recorded on the meter. Grip strength was measured in each animal six successive times, and the average of the values for each mouse was used to calculate muscle strength, normalized to mouse body weights. Statistical analysis was performed using one-way ANOVA (followed by Tukey’s honest significant difference post-hoc test for multiple comparisons), using Graphpad Prism 7 software (GraphPad Software, La Jolla California USA). Data were considered significant for *p* < 0.05. Data are expressed as mean ± SEM. n = 4–18 biological replicates per group.

### Novel object recognition behavioral test

Mice were tested as previously described^81,82^. Mice were acclimated in the testing room 30 min prior to the testing. Novel object testing was performed in the same apparatus used for open-field testing. One day before testing (Day 0), animals were habituated for 5 min, 3 times a day for adaptation. For familiar trial (Day 1), mice were placed in the open arena and allowed to explore for 1 min. Mice were then briefly returned to their home cage while the arena was cleaned, and two identical objects are placed into the field. Mice were given 10 min of object-exploration time to investigate the arena. After this exposure trial, mice were returned to their home cage, and the arena and objects were cleaned with 70% ethanol to remove all olfactory cues. For the novel object test trial (Day 2), one of the familiar objects was replaced with a novel object, and mice were re-introduced to the arena after 24 h. Mice were given 10 min of object-exploration time to investigate the arena. To quantify the amount of time the mice spent exploring the novel object, a ratio was computed whereby the total time spent exploring the novel object was divided by the total time spent exploring both the novel and familiar objects. Behavior was recorded using Any-maze software and later scored offline by trained scorers blind to experimental groups. Statistical analysis was performed using one-way ANOVA (followed by Kruskal–Wallis non-parametric test for multiple comparisons), using Graphpad Prism 7 software (GraphPad Software, La Jolla California USA). Data were considered significant for *p* < 0.05. Data are expressed as mean ± SEM. n = 7–25 biological replicates per group.

### Metabolic treadmill behavioral test

Mice were tested as previously described^81,82^. Mice were weighted and acclimated in the testing room 30 min prior to the testing. Before each testing session, Oxymax software (Columbus Instruments, Columbus, OH, USA) and open circuit indirect calorimetry treadmills (Metabolic Modular Treadmill, Columbus Instruments, Columbus, OH) were calibrated and checked for hardware malfunctions according to manufacturer instructions. Prior to calibration, sample pump was turned on with flow indicator showing flow set at 4–5 LPM. Pressure reading was set at ~ 800 mmHg and gas tank output pressure was set at 5–10 psi. Gas calibration was performed and adjusted when necessary, using the GAIN and FINE knobs to set reading at 0.50% CO_2_ and 20.5% O_2_. Drierite (Calcium Sulfate with Indicator, Sigma-Aldrich; St. Louis, MO, USA) was changed constantly to maintain accurate gas readings and to assure that moisture accumulating during testing could properly be absorbed. During experiments, system sample pump maintained a constant sample flow reading of 0.63 L/min and sample drier a purge gas flow reading of 1.5 L/min. Mice were placed in the chamber with 20° incline and with shock grid supplying with small current (3 Hz and 1.5 mA). We used incremental treadmill protocol for testing, which consisted of 7 stages: stage 1: 2.5 m/min for 3 min, stage 2: 5 m/min for 3 min, stage 3: 10 m/min for 2 min, stage 4: 15 m/min for 2 min, stage 5: 20 m/min for 2 min, stage 6, 25 m/min for 2 min, and stage 7: 30 m/min for 2 min. Stage 7 can be continued to exhaustion by increasing the speed by 2.5 m/min for 2 min. Oxymax computer software collected gas concentrations and flow to calculate oxygen consumption (VO2), carbon dioxide expiration (VCO2), and RER (VCO2/VO2) from the treadmill every 15 s. Maximum run speed (meter/min) and time until exhaustion (min) were also recorded. Statistical analysis was performed using one-way ANOVA (followed by Tukey’s honest significant difference post-hoc test for multiple comparisons), using Graphpad Prism 7 software (GraphPad Software, La Jolla California USA). Data were considered significant for *p* < 0.05. Data are expressed as mean ± SEM. n = 2–8 biological replicates per group.

### Oxygen consumption measurements

Oxygen consumption measurements were carried out using a Seahorse Xfe96 Analyzer as previously described^52,80^. The residual oxygen consumption after 1 µM Piericidin A and Antimycin A combined injection was measured and subtracted from oxygen consumption measurements to determine the basal mitochondrial oxygen consumption. Protocol for extraction and culture of early post-natal cerebellar granule neurons was adapted from previously published methods^83^. Prior to experimentation, mouse genotype was determined, and tissue culture plates were coated with poly-D-lysine (Fisher) at 500 µg/mL. The following day the developing cerebellum of individual early postnatal mice was removed, and meninges were removed under a dissecting microscope. Next, tissue was incubated at 37 °C for 15 min in Papain from the Papain Dissociation System Kit (Worthington) combined with 1 mg/mL of DNAse I. Tissue was then triturated using P1000 pipette tips pre-coated with serum and the suspension was allowed to settle for 30 s to 1 min to allow large undissociated pieces to settle. Using serum-coated pipette tips, cells remaining in suspension were transferred to a new centrifuge vial and centrifuged at 200xg for 5 min. Cell pellets were resuspended in Minimum Essential Media + albumin-ovomucoid inhibitor. Resuspended cells were carefully layered over an additional 1 mL of albumin-ovomucoid inhibitor solution in a new centrifuge vial and centrifuged at 70×*g* for 6 min, pelleting dissociated cells and leaving membrane fragments and lipid at the surface. Next, the cells were resuspended, passed through a 70-micron filter, and plated on pre-plating plates in Neurobasal A medium supplemented with 10% serum, glutamax, antibiotic/antimycotic, and KCl. Cells were allowed to settle for 20 min. After incubation, granule neurons and neuron progenitors were dislodged with gentle tapping while larger glia remained adhered. The pre-plating step for glial removal was repeated. Finally, an enriched population for cerebellar granule neurons was resuspended in serum-free media containing Neurobasal A Medium supplemented with 2% B-27, glutamax, antibiotic/antimycotic, and KCl and plated directly onto poly-D-lysine-coated Seahorse plates and cultured for 5 days prior to oxygen consumption analysis. n = 5–7 biological replicates per group.

## Supplementary Information

Supplementary Figures.

Supplementary Information 2.

Supplementary Information 3.

## Data Availability

The datasets generated during the current study are freely available. RNA sequencing data is available on GEO (acquisition number GSE190689).
