# Author Correction: Reduced expression of mitochondrial complex I subunit *Ndufs2* does not impact healthspan in mice

**DOI:** 10.1038/s41598-022-12069-9

**Published:** 2022-05-17

**Authors:** Gregory S. McElroy, Ram P. Chakrabarty, Karis B. D’Alessandro, Yuan-Shih Hu, Karthik Vasan, Jerica Tan, Joshua S. Stoolman, Samuel E. Weinberg, Elizabeth M. Steinert, Paul A. Reyfman, Benjamin D. Singer, Warren C. Ladiges, Lin Gao, José Lopéz‑Barneo, Karen Ridge, G. R. Scott Budinger, Navdeep S. Chandel

**Affiliations:** 1 Department of Medicine Division of Pulmonary and Critical Care Medicine, Northwestern University Feinberg School of Medicine, Chicago, IL USA; 2 Department of Pathology, Northwestern University Feinberg School of Medicine, Chicago, IL USA; 3 Department of Comparative Medicine, University of Washington School of Medicine, Seattle, WA USA; 4 Instituto de Biomedicina de Sevilla (IBiS), Hospital Universitario Virgen del Rocío, CSIC, Universidad de Sevilla, Seville, Spain; 5 Departamento de Fisiología Médica Y Biofísica, Facultad de Medicina, Universidad de Sevilla, Seville, Spain; 6 Centro de Investigación Biomédica en Red Sobre Enfermedades Neurodegenerativas (CIBERNED), Madrid, Spain; 7 Department of Biochemistry and Molecular Genetics, Northwestern University Feinberg School of Medicine, Chicago, IL 60611 USA

Correction to: *Scientific Reports* 10.1038/s41598-022-09074-3, published online 25 March 2022

Yuan-Shih Hu and Karen Ridge were omitted from the author list in the original version of this Article.

The Author contributions section now reads:

“Conceptualization, methodology, G.S.M., S.E.W., P.A.R., B.D.S., W.C.L., K.R., L.G., J.L.B., G.R.S.B., N.S.C. Supervision, funding acquisition: G.R.S.B., N.S.C. Investigation: G.S.M., R.P.C., K.B.D., Y.H., K.V., J.T., J.S.S., S.E.W., E.M.S., W.C.L. Resources: L.G., J.L.B., W.C.L. Project administration, writing, and editing: G.S.M., R.P.C., K.V., G.S.S.B., N.S.C.”

Additionally, the Acknowledgments section contained errors.

“We thank the following members of the Northwestern University Department of Medicine, Division of Pulmonary and Critical Care for technical support and data analysis, Jennifer Yuan-Shih Hu, Luzivette Robles Cardona, Kathryn Helmin, Nikita Joshi, Francisco J Gonzalez-Gonzalez, Satoshi Watanabe, Luisa Morales-Nebreda, Ziyan Lu, Hiam Abdala-Valencia, and Peng Gao. Funding for this project was provided by the following NIH grants: NIH2PO1HL071643-11A1, NIH1R35CA197532-01 to N.S.C.; NIH1PO1AG049665-01 to G.R.S.B and N.S.C.; NIH/NCI T32CA09560 to G.S.M.; NHLBI F32HL136111 to P.A.R.; P.A.R. was supported by an American Thoracic Society/Boehringer Ingelheim Partner grant; R.P.C was supported by a Northwestern University Pulmonary and Critical Care Department Cugell predoctoral fellowship.”

now reads:

“We thank the following members of the Northwestern University Department of Medicine, Division of Pulmonary and Critical Care for technical support and data analysis, Luzivette Robles Cardona, Kathryn Helmin, Nikita Joshi, Francisco J Gonzalez-Gonzalez, Satoshi Watanabe, Luisa Morales-Nebreda, Ziyan Lu, Hiam Abdala-Valencia, and Peng Gao. Funding for this project was provided by the following NIH grants: NIH2PO1HL071643-11A1, NIH1R35CA197532-01 to N.S.C.; NIH1PO1AG049665-01 to G.R.S.B and N.S.C.; NIH/NCI T32CA09560 to G.S.M.; NIH P01 AG049665, P01 HL071643, P01 HL154998 to K.R.NIH/NCI T32CA09560 to G.S.M.; NHLBI F32HL136111 to P.A.R.; P.A.R. was supported by an American Thoracic Society/Boehringer Ingelheim Partner. We thank the following core facilities at Northwestern: Pulmonary NextGen Sequencing Core, RHLCCC Metabolomics Core, RHLCCC Flow Cytometry Core Facility. Histology services were provided by the Northwestern University Mouse Histology and Phenotyping Laboratory which is supported by NCI P30-CA060553 awarded to the RHLCC. We thank the Center for Comparative Medicine staff. We thank Elizabeth Bartom for the use of the Ceto pipeline.”

The original Article has been corrected.

