# Supplementary material for: Reduced expression of mitochondrial complex I subunit Ndufs2 does not impact healthspan in mice
Source: Sci Rep. 2022 Mar 25;12:5196. doi: 10.1038/s41598-022-09074-3 (PMC8956724; doi:10.1038/s41598-022-09074-3)
Supplement: Supplementary file 1 — Supplementary Figures. [file 41598_2022_9074_MOESM1_ESM.pdf]

Supplemental Figures

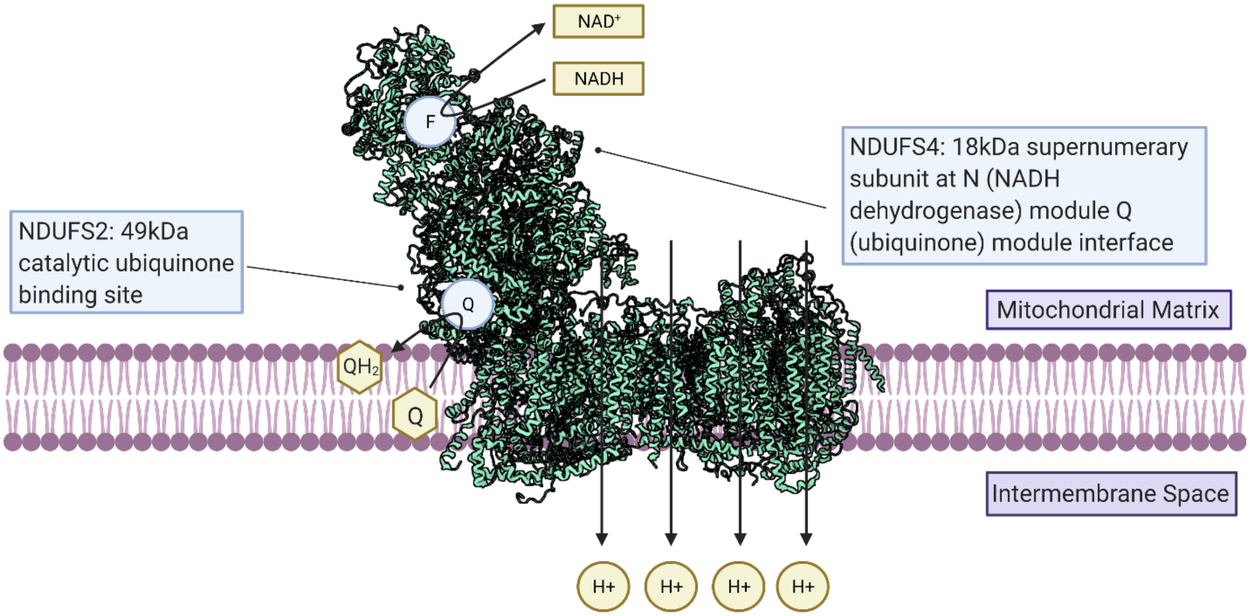

Figure S1. Complex I Schematic. *Ndufs2* encodes the 49kDa catalytic subunit of complex I and is therefore necessary for any enzymatic activity and assembly. *Ndufs4* encodes an 18kDa accessory subunit that is involved in complex assembly and stability and residual function of complex I is seen in tissues without NDUFS4. Created with BioRender.com.

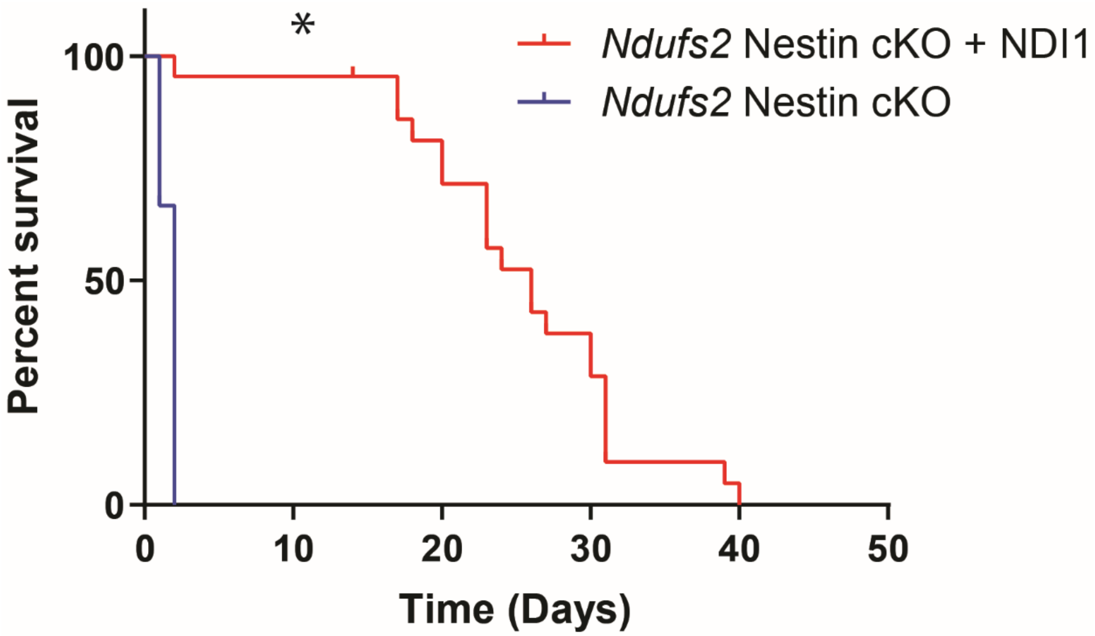

Figure S2. *Ndufs2* deficiency in the brain is lethal and is partially rescued by expression of a yeast alternate NADH dehydrogenase. Survival of Nestin cre mediated *Ndufs2* deficient mice with or without expression of the yeast alternate NADH dehydrogenase NDI1. Log-rank (Mantel-Cox) test *p<0.0001 n=22 cKO=NDI1 , n= 6 cKO.

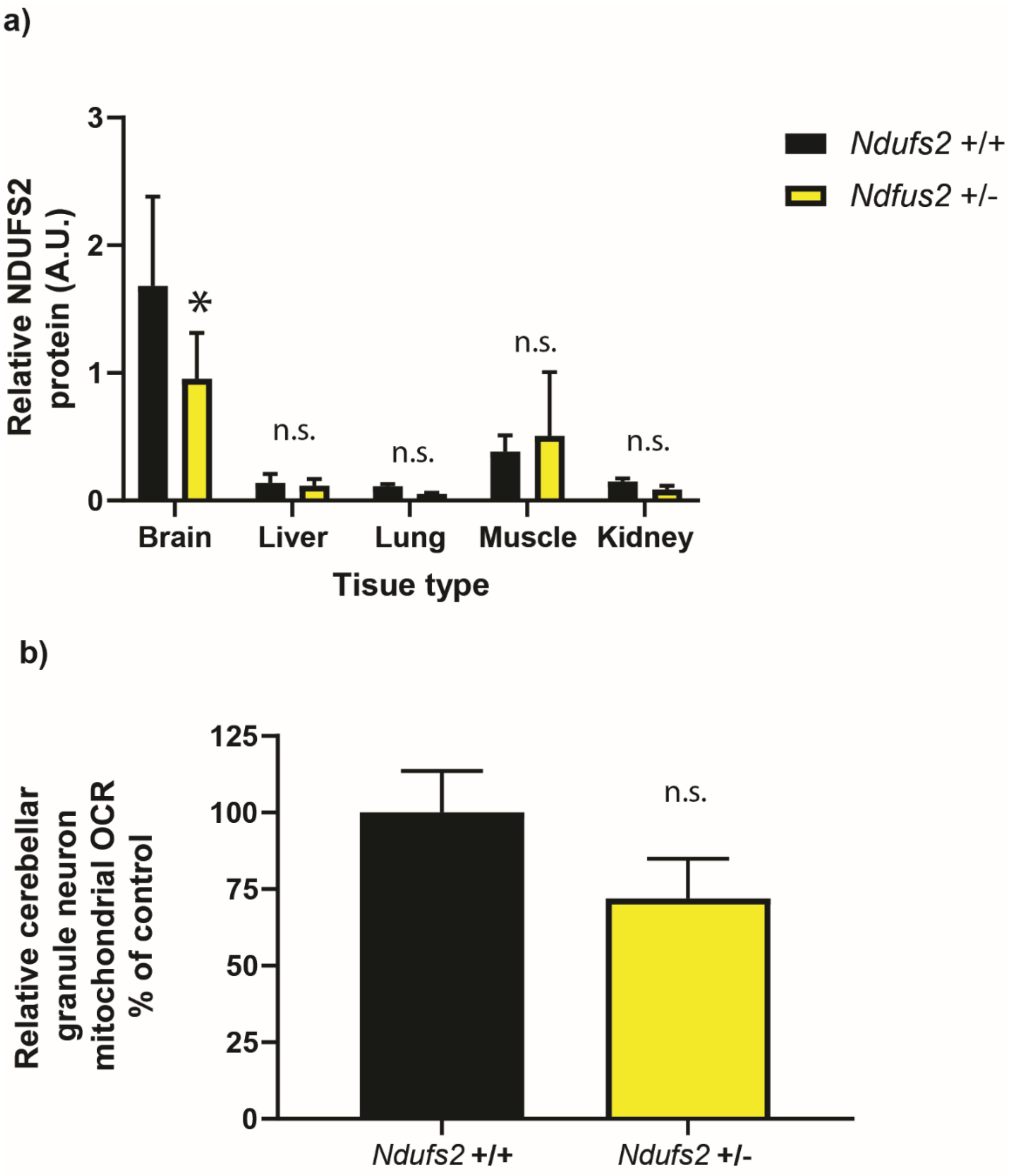

Figure S3. NDUFS2 quantification and neuronal basal OCR

(a) Relative NDUFS2 protein abundance in whole tissue. 2-way ANOVA column factor p= 0.1542 n=3-4 Sidak's multiple comparisons test *p<0.05

(b) Cerebellar granule neuron oxygen consumption rate. Unpaired T-test p= 0.1817. n=5-7. n.s. not significant.

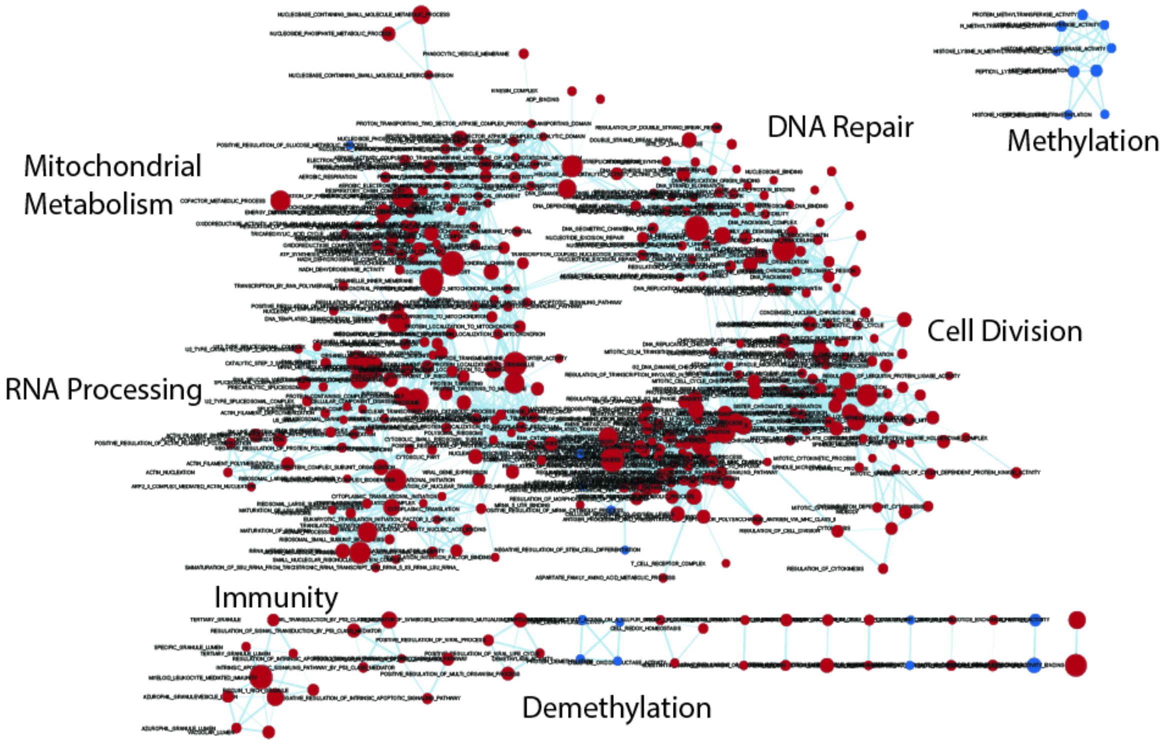

Figure S4. Aged Treg Gene Ontology Enrichment Mapping. Network analysis for gene ontology biological process gene set enrichment analysis of differential gene expression between 20-month-old regulatory T cells from *Ndufs2*$^{+/+}$ versus *Ndufs2*$^{+/-}$ mice. Key networks are identified. Red represents gene sets enriched in *Ndufs2*$^{+/+}$ and blue represents gene sets enriched in *Ndufs2*$^{+/-}$. Circle size represents gene set size. Adjusted P value threshold 0.05.

a)

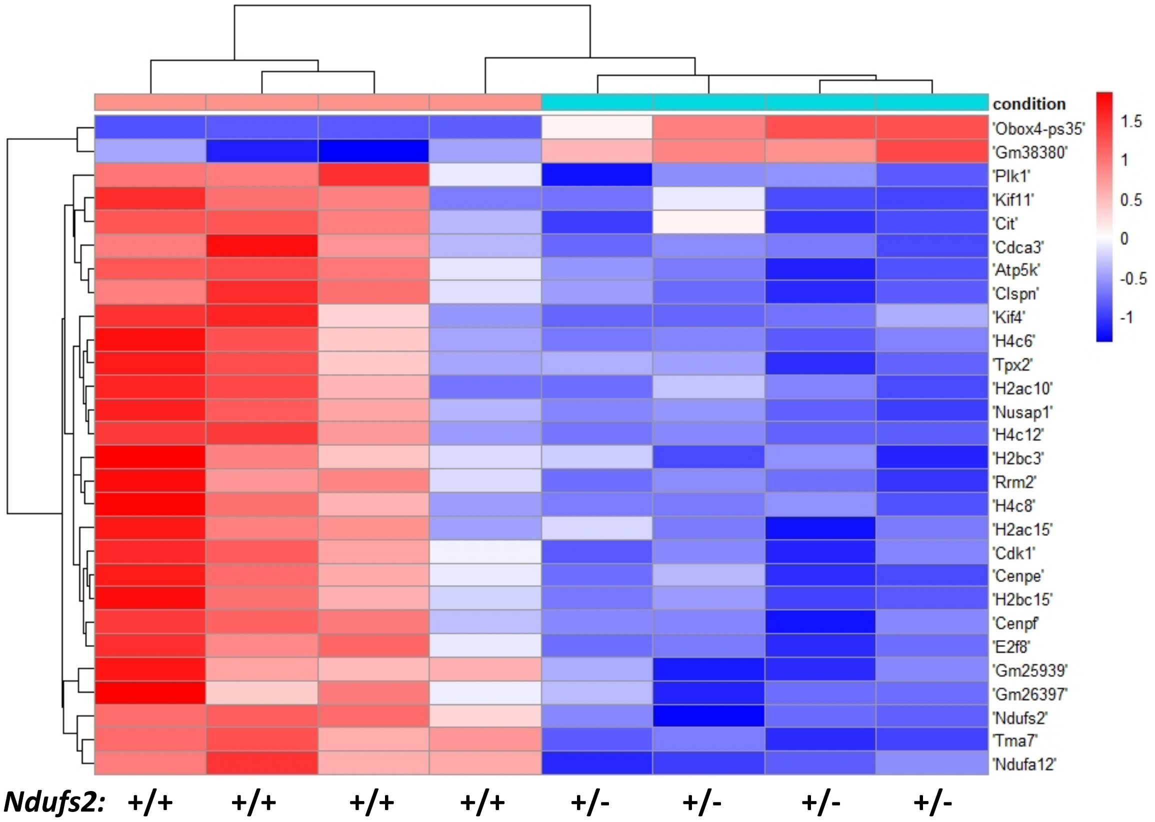

b)

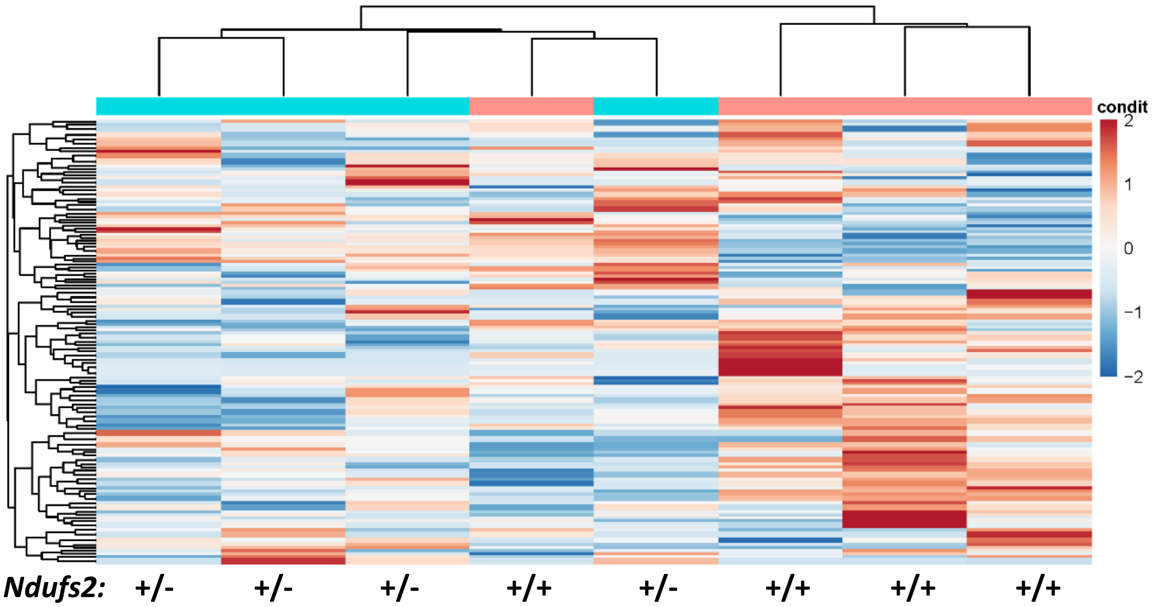

Figure S5. Aged Treg transcriptomics

a) Heat map of the most dysregulated in genes in aged Tregs from *Ndufs2+/+* vs *Ndufs2+/-* mice with a threshold of adjusted P value of 0.05 and log2 fold change >1. Z scores calculated by row

and represented by blue-red spectrum, unsupervised hierarchical clustering of columns and rows.

b) Heat map of the integrated stress response genes in aged Tregs from *Ndufs2+/+* vs *Ndufs2+/-* mice. Z scores calculated by row and represented by blue-red spectrum, unsupervised hierarchical clustering of columns and rows.

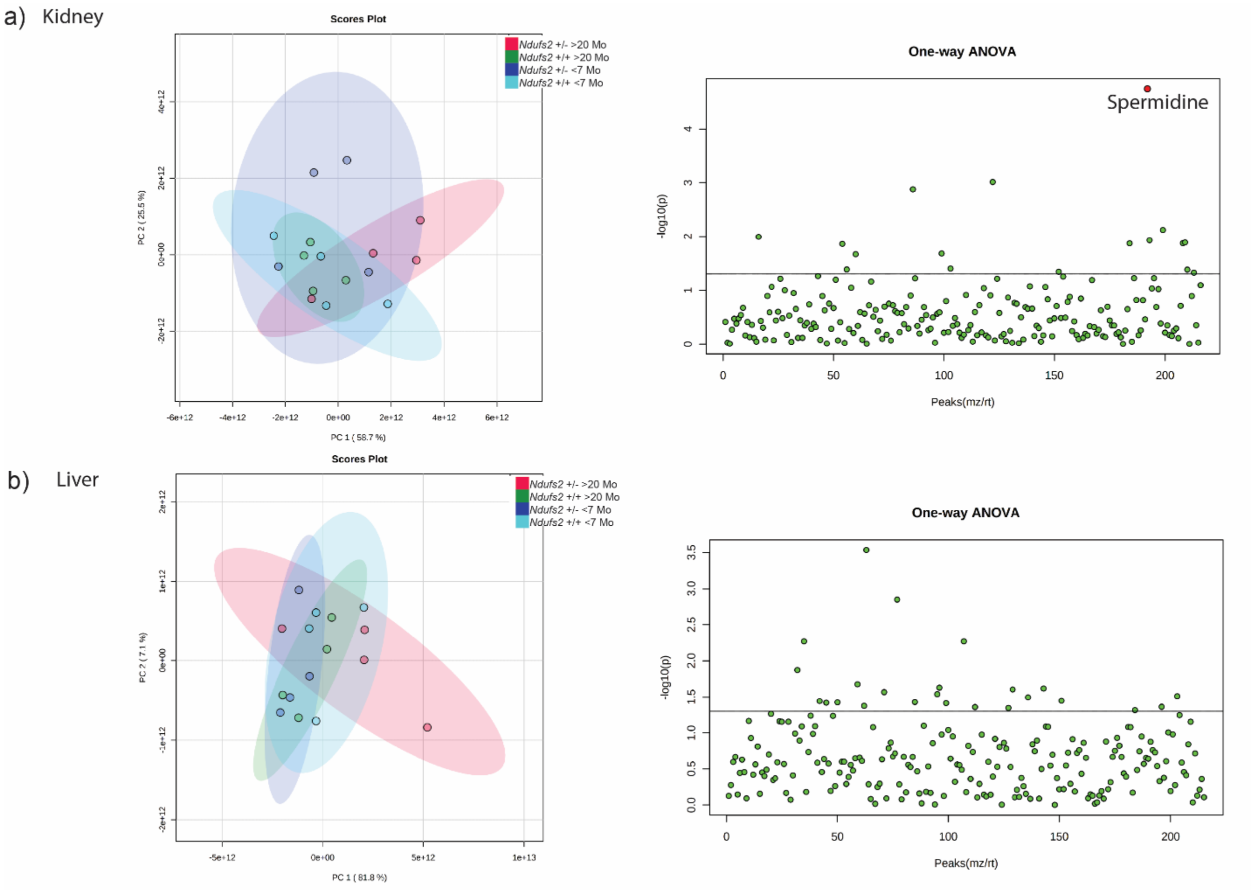

Figure S6. Kidney and Liver metabolite levels

a) Kidney tissue metabolite measurements. Principal component analysis scores plot, one-way ANOVA results, with adjusted P value < 0.05 highlighted in red (spermidine). Young mice <7 months vs aged mice >20 months age matched by genotype. n=4.

b) Liver tissue metabolite measurements. Principal component analysis scores plot, one-way ANOVA results, with adjusted P value < 0.05 no significant metabolites. Young mice <7 months vs aged mice >20 months age matched by genotype. n = 4.

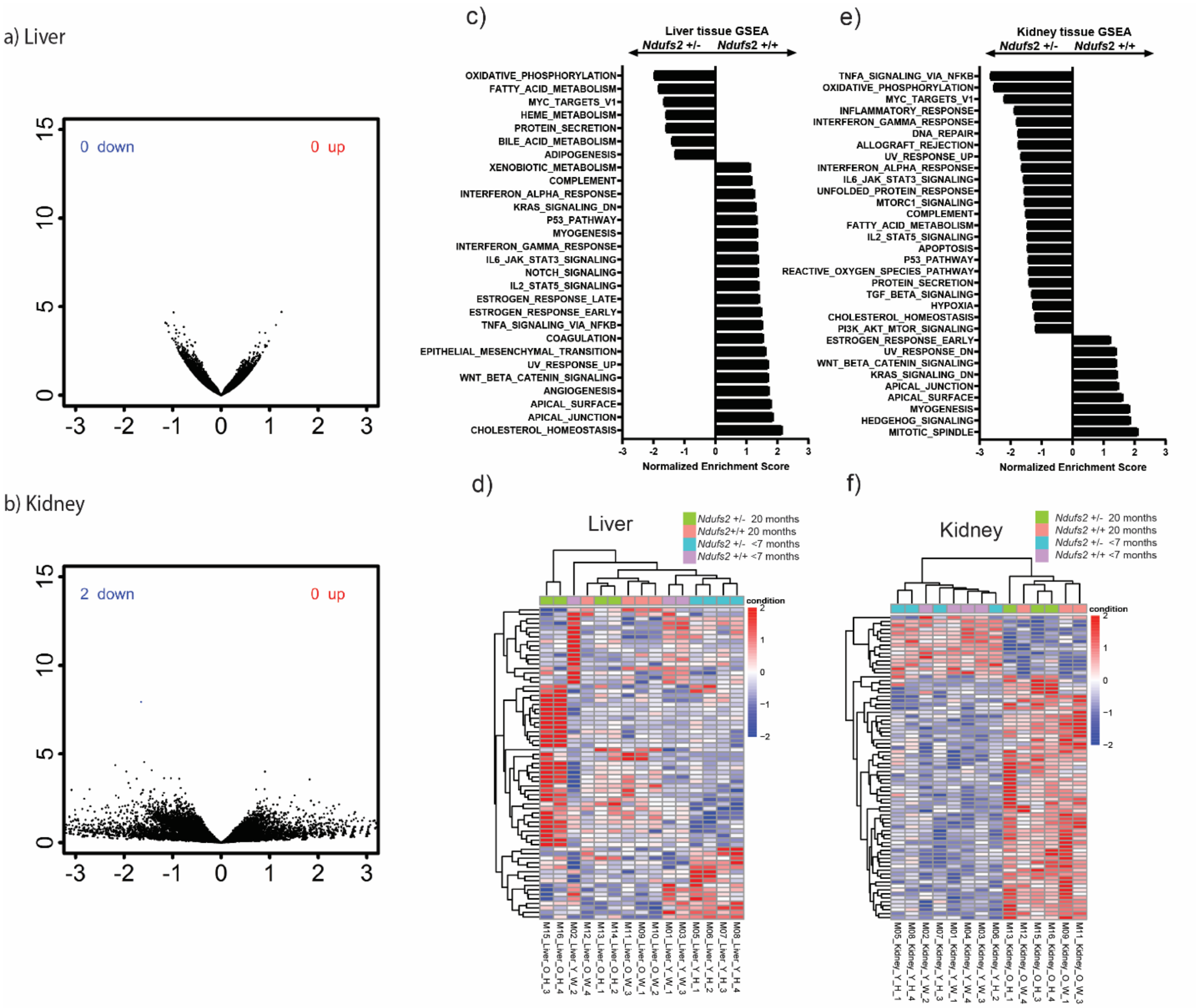

Figure S7. Kidney and Liver transcriptomics

a) Differential gene expression analysis in liver tissue at 20 months of age from *Ndufs2*$^{+/+}$ vs *Ndufs2* $^{+/-}$ mice. n=4. Volcano plot FDR cutoff of 0.05.

b) Differential gene expression analysis in kidney tissue at 20 months of age from *Ndufs2*$^{+/+}$ and *Ndufs2* $^{+/-}$ mice. n=3. Volcano plot FDR cutoff of 0.05.

c) GSEA in liver tissue at 20 months of age from *Ndufs2*$^{+/+}$ vs *Ndufs2* $^{+/-}$ mice. n=4 GSEA MSigDB hallmark gene sets with FDR cutoff of 0.25.

d) Differential gene expression analysis in liver tissue from young (<7 months) and old (20 months) *Ndufs2*$^{+/+}$ and *Ndufs2* $^{+/-}$ mice. 69 individual genes met ANOVA-like test FDR cutoff of 0.05. Unsupervised clustering of columns and rows.

e) GSEA in kidney tissue at 20 months of age. n=3. GSEA MSigDB hallmark gene sets with FDR cutoff of 0.25.

f) Differential gene expression analysis in kidney tissue from young (<7 months) and old (20 months) *Ndufs2*$^{+/+}$ and *Ndufs2*$^{+/-}$ mice. 77 individual genes met ANOVA-like test FDR cutoff of 0.05. Unsupervised clustering of columns and rows.

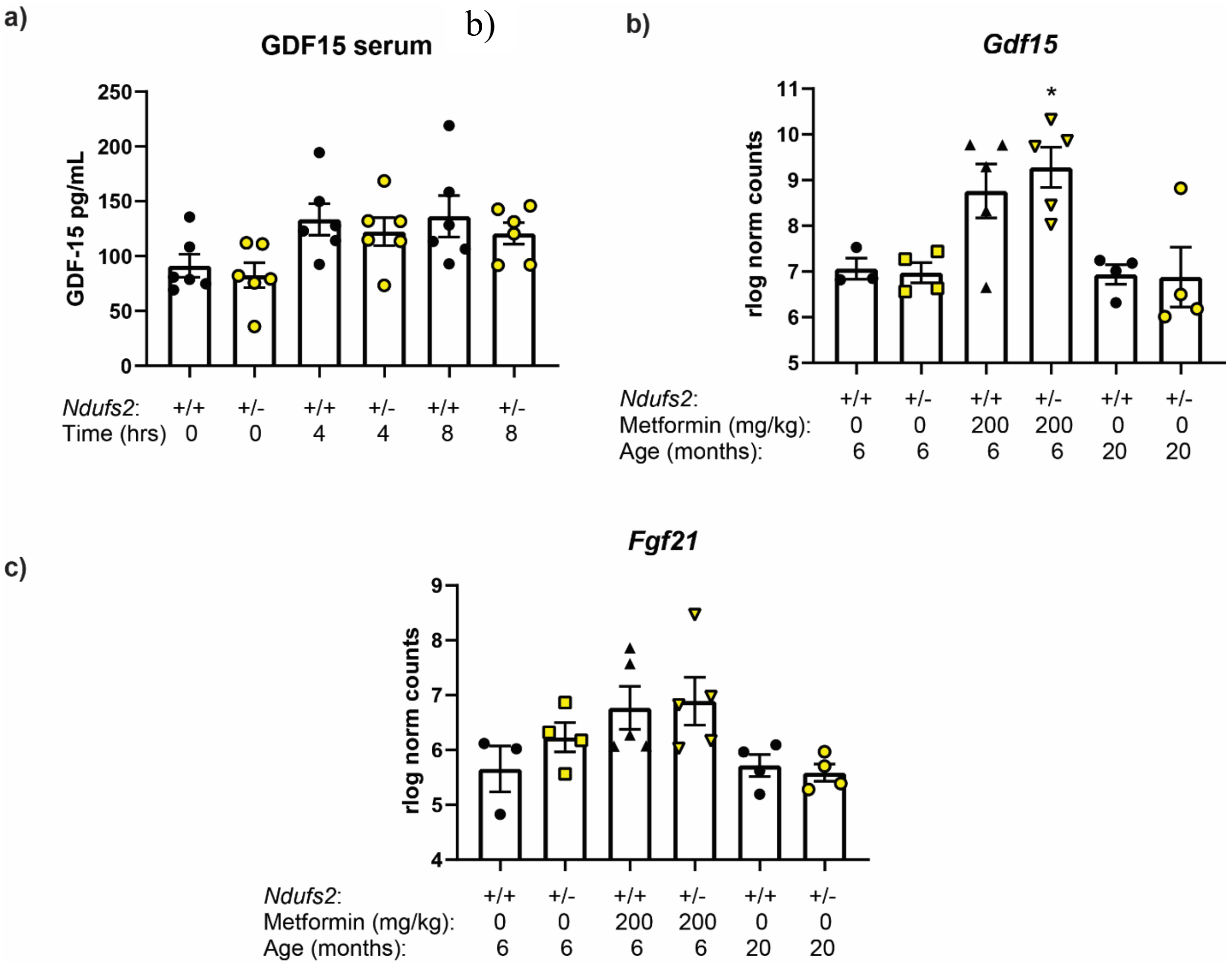

Figure S8. *Gdf15* and *Fgf21* measurement

a) Serum GDF-15 measurements 0, 4, and 8 hours after treatment with 200mg/Kg metformin in *Ndufs2*$^{+/+}$ and *Ndufs2*$^{+/-}$ mice. Ordinary one-way ANOVA p=0.0328, Sidak's multiple comparisons tests by genotype not significant. n=6. Error bars represent standard error.

b) Liver *Gdf15* mRNA from mice with or without 8-hour treatment with 200mg/Kg metformin compared with aged mice (20 months). Regularized log2 normalized counts. Ordinary one-way ANOVA p=0.0022, Tukey's multiple comparisons tests *Ndufs2*$^{+/-}$ with metformin vs *Ndufs2*$^{+/+}$ untreated, *Ndufs2*$^{+/-}$ untreated, 20 month *Ndufs2*$^{+/+}$, and 20 month *Ndufs2*$^{+/-}$ *$p_{adjusted}<0.05$. Tukey's not significant for all other comparisons.

c) Liver *Fgf21* mRNA from young mice with or without 8-hour treatment with 200mg/Kg metformin compared with aged mice (20 months). Regularized log2 normalized counts. Ordinary one-way ANOVA p=0.0296, Tukey's multiple comparisons tests not significant for all comparisons.

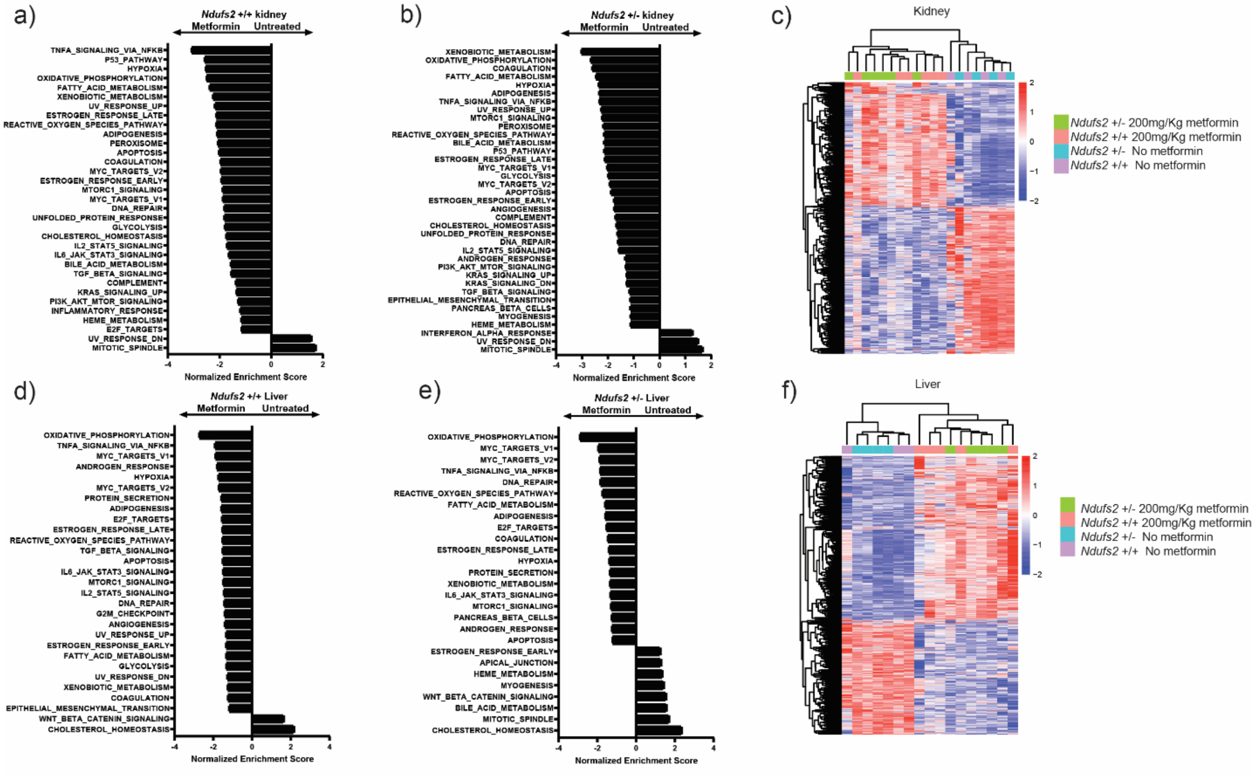

Figure S9. Metformin-induced gene expression changes in liver and kidney at 8 hours

a) *Ndufs2*$^{+/+}$ mouse differential gene expression analysis in kidney tissue from untreated mice versus mice 8 hours after treatment with 200mg/Kg metformin. GSEA MSigDB hallmark gene sets with FDR cutoff of 0.25. n=4-6

b) *Ndufs2*$^{+/-}$ mouse differential gene expression analysis in kidney tissue from untreated mice versus mice 8 hours after treatment with 200mg/Kg metformin. GSEA MSigDB hallmark gene sets with FDR cutoff of 0.25. n=4-6

c) Heatmap of gene expression analysis in mouse kidney tissue with or without 8-hour treatment with 200mg/Kg metformin. ANOVA-like test 1643 significant genes with FDR 0.05. Unsupervised clustering of columns and rows. n=4-6.

d) *Ndufs2*$^{+/+}$ mouse differential gene expression analysis in liver tissue from untreated mice versus mice 8 hours after treatment with 200mg/Kg metformin. GSEA MSigDB hallmark gene sets with FDR cutoff of 0.25. n=3-5

e) *Ndufs2*$^{+/-}$ mouse differential gene expression analysis in liver tissue from untreated mice versus mice 8 hours after treatment with 200mg/Kg metformin. GSEA MSigDB hallmark gene sets with FDR cutoff of 0.25. n=4-5

f) Heatmap of gene expression analysis in mouse liver tissue with or without 8-hour treatment with 200mg/Kg metformin. ANOVA-like test 1550 significant genes with FDR 0.05. Unsupervised clustering of columns and rows. n=3-5.
